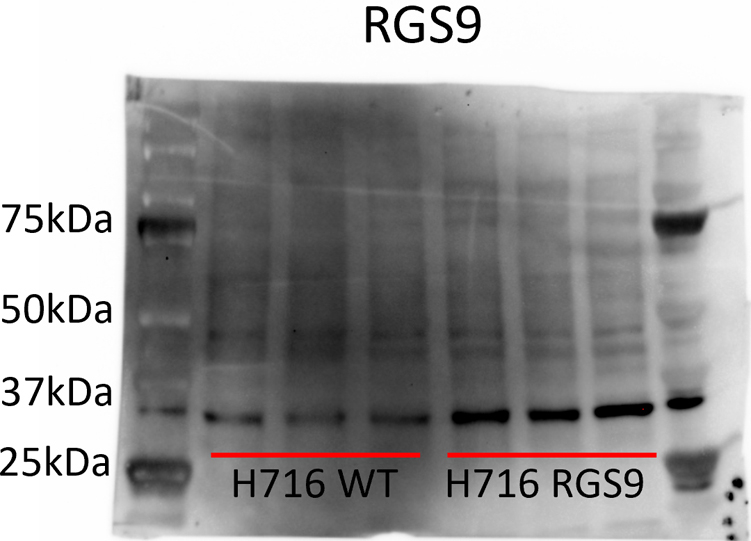# Regulator of G-protein signaling expression in human intestinal enteroendocrine cells and potential role in satiety hormone secretion in health and obesity

**DOI:** 10.1016/j.ebiom.2024.105283

**Published:** 2024-08-13

**Authors:** Alison N. McRae, Alexander L. Ticho, Yuanhang Liu, Maria Laura Ricardo-Silgado, Nothando N. Mangena, Fauzi Feris Jassir, Daniel Gonzalez-Izundegui, Gerardo Calderon, Fariborz Rakhshan Rohakhtar, Vernadette Simon, Ying Li, Cadman Leggett, Daniela Hurtado, Nicholas LaRusso, Andres J. Acosta

**Affiliations:** a Precision Medicine for Obesity Program and Division of Gastroenterology and Hepatology, Mayo Clinic, Rochester, MN, USA; b Division of Biomedical Statistics and Informatics, Mayo Clinic, Rochester, MN, USA; c Center for Individualized Medicine (CIM), Mayo Clinic, Rochester, MN, USA; d Division of Gastroenterology and Hepatology, Mayo Clinic, Rochester, MN, USA; e Division of Endocrinology, Diabetes, Metabolism, and Nutrition Mayo Clinic, Jacksonville, FL, USA

**Keywords:** scRNA-Seq, Obesity, GLP-1, PYY, Enteroendocrine cells, L-cells, RGS

## Abstract

**Background:**

Gut L-type enteroendocrine cells (EECs) are intestinal chemosensory cells that secrete satiety hormones GLP-1 and PYY in response to activation of G-protein coupled receptors (GPCRs) by luminal components of nutrient digestion and microbial fermentation. Regulator of G-protein Signaling (RGS) proteins are negative regulators of GPCR signaling. The expression profile of RGS in EECs, and their potential role in satiety hormone secretion and obesity is unknown.

**Methods:**

Transcriptomic profiling of RGS was completed in native colonic EECs was completed using single-cell RNA sequencing (scRNA-Seq) in lean and obesity, and human jejunal EECs with data obtained from a publicly available RNAseq dataset (GSE114853). RGS validation studies were completed using whole mucosal intestinal tissue obtained during endoscopy in 61 patients (n = 42 OB, n = 19 Lean); a subset of patients’ postprandial plasma was assayed for GLP-1 and PYY. *Ex vivo* human intestinal cultures and *in vitro* NCI–H716 cells overexpressing RGS9 were exposed to GLP-1 secretagogues in conjunction with a nonselective RGS-inhibitor and assayed for GLP-1 secretion.

**Findings:**

Transcriptomic profiling of colonic and jejunal enteroendocrine cells revealed a unique RGS expression profile in EECs, and further within GLP-1+ L-type EECs. In obesity the RGS expression profile was altered in colonic EECs. Human gut *RGS9* expression correlated positively with BMI and negatively with postprandial GLP-1 and PYY. RGS inhibition in human intestinal cultures increased GLP-1 release from EECs *ex vivo*. NCI–H716 cells overexpressing *RGS9* displayed defective nutrient-stimulated GLP-1 secretion.

**Interpretation:**

This study introduces the expression profile of RGS in human EECs, alterations in obesity, and suggests a role for RGS proteins as modulators of GLP-1 and PYY secretion from intestinal EECs.

**Funding:**

AA is supported by the NIH(C-Sig P30DK84567, K23 DK114460), a Pilot Award from the Mayo Clinic Center for Biomedical Discovery, and a Translational Product Development Fund from The Mayo Clinic Center for Clinical and Translational Science Office of Translational Practice in partnership with the 10.13039/100018188University of Minnesota Clinical and Translational Science Institute.

**Research in context**

**Evidence before this study**

Hormone-secreting gut enteroendocrine cells (EECs) are specialized for luminal sensing and express nutrient-activated GPCRs. Activation of these receptors by ingested nutrients results in secretion of enteroendocrine hormones such as GLP-1 and PYY, regulating appetite, food intake, and glucose homeostasis. Regulator of G-protein Signaling (RGS) proteins represent a major mechanism for the negative regulation of GPCR signaling. Tissue-specific expression of RGS is essential to the regulation of various critical processes, many already well established in the cardiovascular, immune, and central nervous system. However, there are no published studies describing the expression profile of the RGS family in human EECs, and the role RGS may play in EEC functionality and its aberrations in disease states like obesity remains unknown.

**Added value of this study**

Transcriptomic profiling of human colonic EECs using scRNAseq, and analysis of a jejunal EEC transcriptomic database identified expression of the RGS family in EECs, and further revealed an altered colonic EEC RGS transcriptomic landscape in obesity. In EECs, RGS, specifically RGS9, may modulate the secretion of nutrient-stimulated GPCR-mediated GLP-1 and PYY from gut L cells, and this system may serve as a potential target for the pharmacological treatment of obesity.

**Implications of all the available evidence**

Many pharmacological attempts have been made to simulate the physiological effects of L-cell satiety hormone peptides GLP-1 and PYY as a treatment for obesity. Targeting EEC-specific RGS proteins may present new beneficial applications to therapeutic strategies in GPCR-based drug discovery for obesity. A pharmacological approach to stimulating maximal endogenous secretion of satiety hormones may include the combination of GPCR-agonists coupled with EEC-specific RGS-inhibitors.

## Introduction

Enteroendocrine cells (EECs) are rare hormone-secreting cells sparsely distributed throughout the length of the gastrointestinal (GI) tract, representing approximately 1% of the gut mucosal population.^1^ Collectively, EECs constitute the largest endocrine system in the body, and play a major role in the regulation of metabolic homeostasis. Intestinal EECs are specialized for sensing the GI luminal environment and secrete over 20 hormones in response to luminal factors to influence gastrointestinal secretion and motility, regulate food intake, and glucose homeostasis.^1^ Traditional hormone-based classifications of EECs generally delineate eight distinct subtypes based on their localization along the GI tract, and hormone secretory profile.^2^ However a growing body of evidence suggests the existence of both heterogeneity and plasticity of EEC hormone secretory profiles, and the traditional classification may soon require updating.^3^^,^^4^ To sense the wide range of potential luminal stimuli, EECs express a diverse variety of sensory mechanisms, including G-protein-coupled receptors (GPCRs), nutrient transporters, and ion channels.^5^ The classically define L-type EECs are most abundant in the ileum and colon and predominately secrete satiety-inducing hormones glucagon-like peptide 1 (GLP-1) and peptide-YY3-36 (PYY) after a meal in response to activation of their surface GPCRs by luminal components of nutrient digestion and microbial fermentation, which function to retard gastric and small bowel transit, maintain glucose homeostasis, and reduce food intake and appetite.^6^^,^^7^

The systemic impact of EEC-secreted gut hormones on control of food intake, appetite, and glucose homeostasis has led to considerable investigation into their role in the pathogenesis and treatment of metabolic disorders including obesity.^1^ In previous studies, levels of satiety hormones GLP-1 and PYY have been found to be lower in obesity compared to normal weight controls, increased 10-fold after bariatric surgery, and decreased with diet-induced weight loss.^8^, ^9^, ^10^, ^11^, ^12^, ^13^ Gut satiety hormones additionally represent powerful therapeutic targets. Indeed, long-acting GLP-1 analogs are available clinically for treatment of T2D and obesity.^14^ Furthermore, stimulation of endogenous secretion from EECs using small molecule GPCR-agonists represents an appealing therapeutic strategy for these and other metabolic disorders.

In EECs, activation of chemosensory GPCRs and their downstream effectors orchestrate metabolism, digestion, and food intake. In other tissues, GPCR signaling is tightly regulated by various mechanisms. Regulator of G-protein Signaling (RGS) proteins, a family of 20 canonical proteins that serve as key negative regulators of GPCR signaling, function as GTPase-activating proteins (GAPs) to heterotrimeric G-proteins, leading to the rapid termination of G-protein signaling.^15^ Tissue-specific expression of RGS is essential to the regulation of various critical processes, many already well established in the cardiovascular, immune, and central nervous system.^16^, ^17^, ^18^ As RGS proteins represent a major mechanism for the negative regulation of GPCR signaling, we hypothesized that EECs, which heavily rely on GPCR signaling to carry out their main luminal sensing functionality, also employ RGS proteins to coordinate GPCR activation and deactivation within the cell. However, there are no published studies describing the expression profile or function of the RGS family in human EECs.

In the current study, we aimed to establish the RGS expression profile of native human intestinal EECs, describe alterations to their transcriptional landscape in obesity, and further explore the physiological role of RGS in GPCR-mediated secretion of GLP-1 from EECs and the functional consequence of its dysregulation.

## Methods

### Participants, tissue collection and hormone testing

The study was approved by the Mayo Clinic Institutional Review Board, and all participants gave written informed consent following thorough explanation of the study details. Studies were performed at the Mayo Clinic Clinical Research Trials Unit (CRTU) after an 8-h fasting period. Endoscopic mucosal tissue was collected from a total of 61 participants (lean n = 19; obesity n = 42) after receiving a tap water enema during a flexible sigmoidoscopy, or during routine colonoscopy with ileal intubation (Table 1, “Complete Cohort”). During the procedure, 8–16 mucosal biopsies were obtained from the colon and/or ileum. Tissue was then processed for appropriate downstream applications, as described below.

**Table 1:** Patient Demographics for complete cohort and subcohorts: scRNA-Seq discovery cohort and validation cohort.

Cohort	Parameter	Lean	Obesity	p-value
Complete cohort	N	19	42	
	Age (years)	39 ± 13.1	43 ± 11.7	0.26
	Female N (%)	11 (58)	25 (60)	
	BMI (kg/m^2^)	23.1 ± 1.9	39.9 ± 6.9	0.0001
scRNA-Seq^a^	N	4	5	–
	Age (years)	28 ± 1.4	44 ± 4.9	0.001
	Female N (%)	3 (75)	4 (80)	–
	BMI (kg/m^2^)	23.0 ± 2.9	35.4 ± 2.1	0.0001
Colonic RGS hormone associations^a^	N	16	31	–
	Age (years)	40 ± 13.2	39 ± 9.9	0.84
	Female N (%)	9 (56)	20 (65)	–
	BMI (kg/m^2^)	23.2 ± 1.6	41.4 ± 7.0	0.0001
Validation: single-cell RGS family tissue validation^b^	N	6	9	–
	Age (years)	53 ± 4.0	58 ± 4.7	0.06
	Female N (%)	3 (50)	3 (33)	–
	BMI (kg/m^2^)	23.7 ± 1.0	35.5 ± 5.0	0.0001

Data reported as Mean ± SD.

Significance testing used an un-paired student t-test. Significant difference between lean and obesity.

a Denotes studies where colonic biopsies only were collected.

b Denotes studies where both colonic and ileal biopsies were collected.

For determination of plasma postprandial GLP-1 and PYY, participants received a mixture of 63 g glucose in 240 ml of skim milk and a meal with 2 scrambled eggs, 50 g of Canadian Bacon and one slice of bread (∼560 Kcal: 43% carbohydrate, 18% protein, and 40% fat). Plasma samples were collected fasting and postprandial at 15, 45 and 90 min for measurement of GLP-1 (Cat#GLP1T-36HK, Millipore Sigma), and PYY (Cat#PYYT-66HK, Millipore Sigma).

### Single-cell RNA-sequencing and analysis

#### Tissue collection and cryopreservation and FACS-isolation

Mucosal biopsies from the sigmoid colon were collected, cryopreserved, and prepared for scRNA-Seq using FACS-isolation of single, live non-apoptotic cells as previously described.^19^

#### scRNA-Seq and data analysis

We performed all steps following the Chromium 10X Genomics single cell RNA-Sequencing (scRNA-Seq) platform, with a targeted cell capture of 10,000 single cells. We used the Chromium Single Cell 3′ Library & Gel Bead Kit v2 (10X Genomics). In short, all samples and reagents were prepared and loaded into the chip. Then, we ran the Chromium Controller for droplet generation. Reverse transcription was conducted in the droplets. We recovered cDNA through demulsification and bead purification. Pre-amplified cDNA was further subjected to library preparation. Libraries were sequenced on an Illumina Hiseq 4000 for 100 paired-end runs at 1 sample over 2 lanes.

We used 10X Genomics Cellranger Single Cell Software Suite (v3.0.0) to generate FASTQ files, perform alignment to hg38 reference genome, filtering, barcode counting and UMI counting. For subsequent clustering (k-means) and data analysis, we followed the integrated analysis workflow in the Seurat package (v3.1) (https://satijalab.org/seurat/v3.1/integration.html). Genes that were expressed in fewer than 3 cells, cells that expressed fewer than 200 genes and >40% mitochondria genes were excluded for downstream analysis in each sample. Each dataset was normalized using log normalization and scaled for each gene across all cells. All datasets were integrated, scaled, and clustered on the low-dimensional space. Resolution parameter for Seurat was set to 0.3 for all data integrations. Enriched gene markers in each cluster conserved across two conditions were identified with fold change larger than 2, adjusted p-value smaller than 0.05 in both conditions. All clustering and statistical analysis was performed in R (v 3.5.2). Raw count gene expression data from scRNAseq were normalized using Trimmed Mean of M-values (TMM) method from edgeR and converted to transcript per million (TPM).^20^ Cells were categorized into three groups based on the gene expression levels of *GCG*: 1). Cells with positive expression for *GCG* in the Enteroendocrine cluster 15; 2) Cells with no expression for *GCG* in the enteroendocrine cluster 15; 3) Cells in other clusters. Average gene expression levels were computed for each group.

### Analysis of GSE114853 RNA-sequencing database

#### Data acquisition and analysis

The “Human enteroendocrine cell transcriptomic profiling” GSE114853 RNAseq dataset and their associated information were obtained from the NCBI Gene Expression Omnibus (GEO). Methodology for the comparative transcriptomic study is fully described as previously published.^21^ Briefly, transcriptomic profiling of 3 cell populations of human jejunum in 11 participants was completed by bulk RNAseq using Illumina HiSeq 4000. The three cell populations included FACS-purified populations of human jejunal enteroendocrine cells: L-type jejunal EECs (GLP1+/CHGA+/SCG2+), non-L-type jejunal EECs (GLP1-/CHGA+/SCG2+), and the third population contained non-EEC jejunal cells (GLP1-/CHGA-/SCG2-). The raw count gene expression data were then normalized using TMM method from. EdgeR and converted to transcript per million (TPM).^20^ Average gene expression levels were computed for each of the three groups.

### Enteroendocrine physiology validation studies

#### Participants

We interrogated the gut hormone expression profile of 47 participants (Table 1, “Colonic RGS Hormone Associations”)within our studies for either mucosal mRNA and protein expression, plasma hormone levels, or both. In a cohort of 15 participants both colonic and ileal biopsies were collected for validation of RGS expression (Table 1, “Validation: Single-cell RGS family tissue validation”). Colonic and ileal mucosal biopsies, as well as postprandial plasma samples were collected as described above. Colonic and ileal mucosal biopsies were either immediately cryopreserved, placed in RNA*later* (Ambion) for subsequent RT-qPCR, or fixed in 10% NBF for immunofluorescence studies.

#### Traditional RT-qPCR

Total RNA was extracted from RNAlater preserved biopsies using the RNeasy Plus Micro Kit (Cat#74034, Qiagen), reverse transcribed into cDNA using the AffinityScript QPCR cDNA Synthesis Kit (Cat#600559, Agilent Technologies), and amplified by real-time quantitative PCR using gene-specific primers (250 nM final concentration; Supplemental Table S1) and performed in the LightCycler 480 II System (Roche Life Sciences) using SYBR green (Cat#172-5270, Bio-Rad) detection. Eukaryotic Elongation Factor 2 (*EEF2*) was used as an endogenous control.^22^^,^^23^ Gene expression was calculated using the 2-ΔΔCt method and presented as normalized gene expression to *EEF2* expression for each sample.

#### Immunofluorescence

Human mucosal colonic biopsies were fixed in 10% NBF overnight. The fixed tissue was incubated in 70% ethanol for 48 h and stored in sterile PBS at 4 °C until embedding. Tissue was embedded in paraffin and slides containing 5 μm FFPE sections were prepared. The sections were deparaffinized and rehydrated through a graded alcohol series, followed by antigen unmasking (Cat#H-3300-250, Vector Laboratories). Tissue was permeabilized with 0.1% Triton X-100 and then blocked with 10% FBS, 1% BSA, 0.1% Tween-20 in PBS for 1 h at room temperature. Sections were probed with primary antibodies, followed by incubation with fluorochrome conjugated secondary antibodies to detect respective primary antibodies. A negative control and a no primary antibody control was used for each antibody. Antibodies used are described in the Supplementary Materials. Slides were mounted with ProLong™ Gold Antifade Mountant with DAPI (Cat#P3693, Life Technologies). Images were taken on a Confocal Microscope (LSM 980 Axio Observer), and probes were excited using 405, 488, and 633 nm laser lines. Resulting images were analyzed with the ZEN software (ZEN 2.1, Zeiss).

#### Generation of primary cultures from human intestinal biopsies

Primary culture of intestinal monolayers, originating from human colon and ileum were generated using previously reported methodologies.^24^^,^^25^ Aliquots (100 μl) were plated into 96-well plates coated with 4 mg/ml Matrigel, and primary cultures were incubated for 4-h at 37 °C in 5% CO^2^.

#### NCI–H716 general cell maintenance

The Human L cell line NCI–H716 was obtained from the American Type Culture.

Collection (CCL-251, ATCC) and maintained at a density of ∼500,000 cells/ml in suspension culture in T25 suspension flasks (5669-0195, USA Scientific) containing RPMI medium (25–506, GenClone) supplemented with 10% FBS, 100 IU/ml penicillin, and 100 μg/ml streptomycin. Cells were kept at 37 °C, 5% CO_2_, until 85% confluent, then split 1:5 and transferred to fresh T25 flasks.

#### Transfection of NCI–H716 cells and generation of stable expressing RGS9 NCI–H716 cells

NCI–H716 cells were cultured in RPMI-1640 medium with 10% (v/v) FBS and grown at 37 °C in 5% CO_2_. A RGS9 construct containing human RGS9-2 (accession number NM_001081955.3) in pcDNA 3.1 expression vector was purchased form GenScript (Clone ID: OHu05606). Transfection of NCI–H716 cells was performed using Lipofectamine 3000 (Invitrogen) following manufacturer's instructions and selected with Geneticin G418 (100 μg/ml, Gibco).

#### NCI–H716 enteroendocrine differentiation

NCI–H716 cells were split into 96-well plates pre-coated with 150 μl per well 4 mg/ml matrigel (354234, Corning), at a density of 100,000 cells per well in 200 μl media containing high glucose DMEM (11965092, Gibco) supplemented with 10% (v/v) FBS, 100 IU/ml penicillin, and 100 μg/ml streptomycin. Cells were maintained for 48 h to allow for enteroendocrine differentiation.

#### RGS inhibition cell culture experiments

Primary human intestinal cultures were washed with Dulbecco's Phosphate-Buffered Saline (DPBS, Cat#21-030-CV, Corning) and exposed to treatments (200 μl, n = 6 wells/treatment) for 2-h. Treatments included: varying doses of RGS-inhibitor CCG-50014 (Cat#10802, Cayman Chemical) alone, or stimulating GPCR-agonists: meat hydrosylate (2% w/v; Cat#70174, Sigma), 1,10-Phenanthroline (500 μM; Cat#131377, Sigma), Sodium Acetate/Propionate (500 μM each; Cat#S8750, P5436, Sigma), taurocholic acid (500 μM; CAT#T4009, Sigma).^26^, ^27^, ^28^, ^29^ All treatments were prepared in DPBS-0.5% BSA (w/v) control media. CCG-50014 inhibitor dosage range was based on dosages previously described to effectively attenuate RGS activity in RGS14 and RGS10 proteins, members of the R12 RGS protein family, most closely related to the R7 RGS family, which includes RGS9.^30^ Cells were incubated at 37 °C for 2 h, after which media was collected and centrifuged (5 min, 1000×*g*, 4 °C) to pellet any cells, and the supernatant was then frozen at −20 °C for subsequent ELISA analysis. Cell viability was measured using the Trypan Blue exclusion test. The active GLP-1 concentration of the media supernatants was determined by ELISA (Cat#EZGLPHS-35 K, EMD Millipore Sigma) according to manufacturer instructions. Active GLP-1 levels were expressed as fold to their respective controls.

Differentiated NCI–H716 cells were thoroughly washed with DPBS and treated for 2 h with DPBS containing 0.5% BSA and either 1) vehicle (1:1000 DMSO and 1:1000 EtOH), CCG-50014 (25 μM), 1,10-phenanthroline (1 mM), or CCG-50014 (25 μM) and 1,10-phenanthroline (1 mM). Samples were processed as above.

### Ethics statement

This study was approved by The Mayo Clinic Institutional Review Board (IRB), Rochester, MN, United States (Protocol numbers: 17-009999, 17-009678, 16-007060,16-008664). All authors had access to the full data, reviewed and revised the manuscript, and gave approval to submit the manuscript for publication.

### Statistics

Data are expressed as mean ± SD unless otherwise stated. Data for differential gene expression in the RNA sequencing datasets were analyzed using the default Seurat package settings, based on the non-parametric Wilcoxon rank sum test. Graphical data from the RNA sequencing datasets are visualized as bar charts, where top horizontal line of bar represents average expression or proportion, and further denoted with exact numbers above individual bars. The nonparametric Spearman correlation analysis was completed to measure the associations between RGS expression and BMI, human plasma PYY AUC and GLP-1 AUC, and fasting colonic *PYY* and *GCG* mRNA expression. Data for nonparametric correlations are expressed as Spearman correlation coefficients (r) and 95% confidence interval (CI), with accompanying regression lines fitted with ordinary least squares. Significance testing comparing groups for relative RGS levels, normalized GLP-1 cell secretion from human intestinal cultures *ex vivo* and NCI–H716 cells *in vitro*, used a two-tailed unpaired Welch's t-test assuming unequal variances, unless otherwise stated. The distribution of datasets were tested for normality using the Shapiro–Wilks test and through the generation of quantile–quantile (Q–Q) plots. Graphical representations of data showing individual values within a group include a vertical error bar line, denoting SD, and a horizontal line denoting mean value. Data were analyzed with the JMP Pro (Version14, JMP Statistical Discovery, LLC) statistical software. Visualization of data in the form of figures was completed using GraphPad Prism (Version 9.3.0, GraphPad Software, LLC).

### Role of the funding source

The funding sources had no role in the study design; in the collection, analysis, and interpretation of data; in the writing of the report; and in the decision to submit the paper for publication.

## Results

### Single-cell RNA-seq study to profile human colonic Mucosa in obesity

We previously described a workflow for the cryopreservation of endoscopically obtained human intestinal mucosal biopsies, subsequent preparation of this tissue to yield highly viable FACS-isolated human intestinal single-cell suspensions compatible with successful library preparation and deep scRNA-Seq.^19^ Using this workflow, we performed scRNA-Seq using the 10X Genomics platform on live FACS-isolated cell samples derived from cryopreserved colonic mucosal biopsies in a single batch. Samples were obtained from 5 participants with obesity [(mean ± SD): age 44 ± 4.9 years old, BMI 35.4 ± 2.1 kg/m^2^, 80% females] and 4 lean (healthy weight controls) participants [(mean ± SD): age 28 ± 1.4 years old, BMI 23 ± 2.9 kg/m^2^, 75% females] (Table 1, “scRNA-Seq”). Following data filtering we analyzed the transcriptomes of an estimated 16,723 single cells of the human gut mucosa, and detected an average of 24,000 genes, 89% mapping to the genome, and in total, 705 million reads, at 127,000 reads per cell.

### Human colonic Mucosa clusters into 20 subsets and identifies an enteroendocrine subset

A clustering analysis of the human colon partitioned cells into 20 transcriptionally distinct subsets (Supplemental Figure S1a). The EEC cluster was identified by conserved expression of Chromogranin A (*CgA*), a hallmark for gut EECs.^6^^,^^31^^,^^32^ The major classically defined EEC subsets present in the human colon were identified as 5HT-expressing EC cells (*TPH1*), GLP-1 (*GCG*) and *PYY*-expressing L cells, *SST*-expressing D cells (Supplemental Figure S1b and c). A total of 117 EECs were identified comprising approximately 0.75% of the total gut mucosa. The EEC cluster comprised an estimated 0.8% (77 cells), and 0.7% (40 cells) of the total mucosal population in lean and obesity, respectively, and no significant difference in mean ratio of EECs was detected between the groups.

### Transcriptomic RGS profile of human EECs and alterations in obesity

**Fig. 1:**
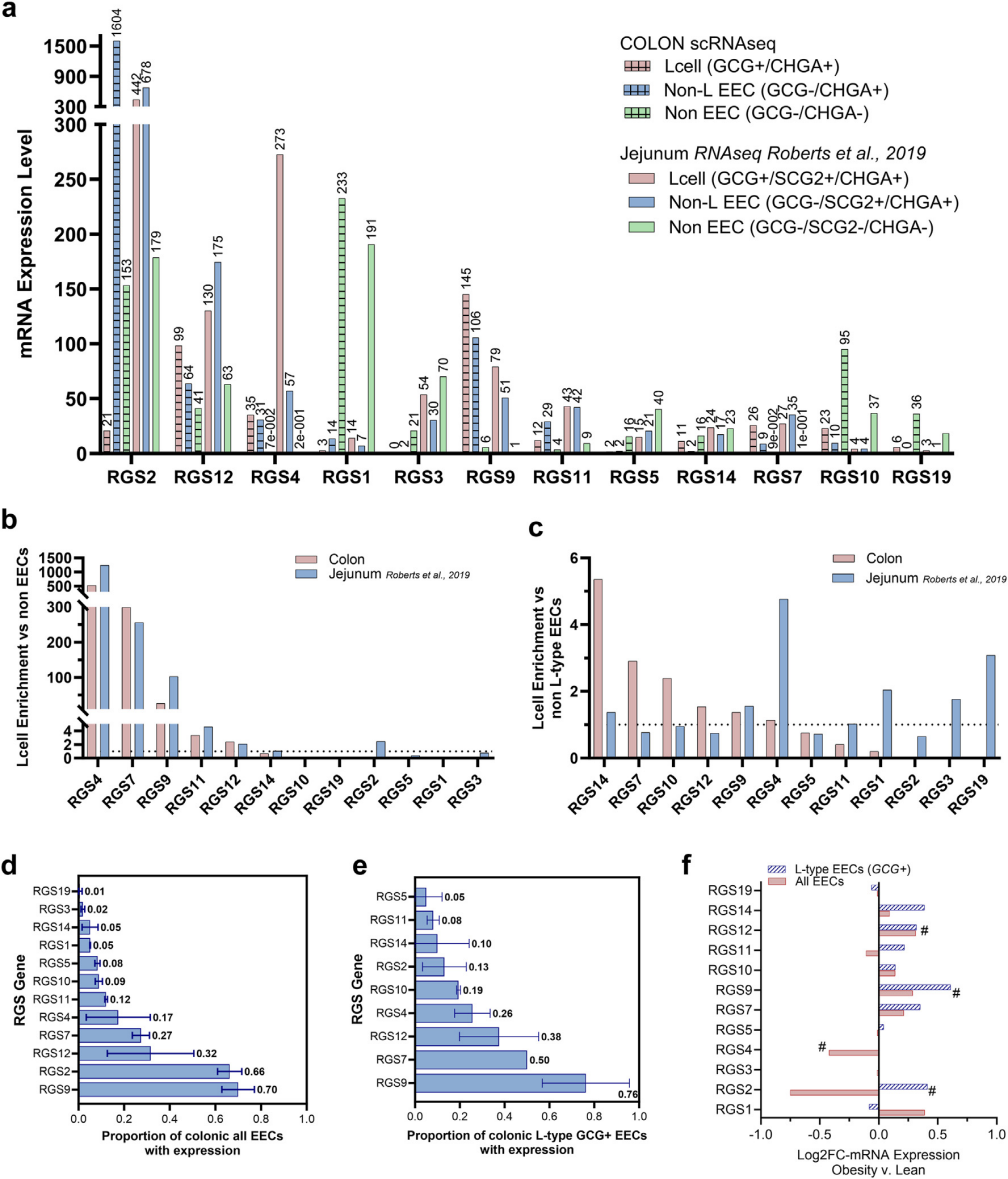
**Transcriptomic profiling of the RGS family in intestinal human EECs and alterations in obesity a)** mRNA Expression levels of RGS family genes within three defined cell types: L-type EECs (red bars), non-L-type EECs (blue bars) and non EECs (green bars) in the human colon profiled from the present scRNAseq study (striped bars), and human jejunum profiled from previous a RNAseq study from *Roberts* et al.*, 2019* (solid bars). Fold-Enrichment values of RGS family mRNA expression within **b)** L-type EECs compared to non-L-type EECs and **c)** L-type EECs compared to non-EECs in human colon profiled from the present scRNAseq study (red bars), and human jejunum profiled from previous a RNAseq study from *Roberts* et al.*, 2019* (blue bars). The average proportion of **d)** all colon EECs within cluster or **e)** L-type cells within EECs expressing detected RGS family genes. **f)** RGS family genes demonstrating differential expression in all EECs within cluster for obesity compared to lean. Bar charts display the Mean value for respective data. Error bar lines denote SD. # denotes physiologically relevant finding defined as RGS genes displaying differential Log2FC in obesity with p < 0.20 in either all EECs or L-type EECs.

We next investigated the RGS transcriptomic profile of native human intestinal EECs from jejunum and colon. Expression of 12 RGS genes were detected in EECs (Fig. 1a). Among *GCG*-expressing EECs, classically defined as L-type EECs, *RGS9*, followed by *RGS12,* were the most highly expressed RGS genes in the colon, whereas *RGS2* followed by *RGS4* was mostly highly expressed in jejunum. In the EECs without *GCG* expression, referred to as non-L-type EECs, *RGS2*, followed by *RGS9* were the most highly expressed in the colon; and *RGS2* and *RGS12* were the mostly highly expressed in jejunum. Expression of *RGS4*, *RGS7*, *RGS9*, *RGS11,* and *RGS12* was enriched in both colonic and jejunal L-type EECs compared to non-EECs (Fig. 1b). Within EECs, Expression of *RGS14*, *RGS7*, *RGS10*, *RGS12,* and *RGS9* was enriched in L-type EECs compared to non-L-type EECs in colon (Fig. 1c). However, in jejunum enrichment of *RGS14*, *RGS9*, *RGS4*, *RGS11*, *RGS1*, *RGS3* and *RGS19* expression was observed in L-type EECs compared to non-L-type EECs. While expression of many RGS genes were detected in EECs, only *RGS2* and *RGS9* were expressed in the majority proportion (>0.50) of colonic all-EECs (0.66 and 0.70, respectively) (Fig. 1d). *RGS9* and *RGS7*, represented the RGS genes displaying expression in a majority proportion of colonic L-type EECs (0.76, 0.50, respectively); similar to all-EECs, RGS9 was again expressed in the greatest proportion of L-type EECs (Fig. 1e).

We next explored transcriptional alterations to RGS expression within colonic EECs in the context of obesity. We considered physiologically relevant hits as RGS genes displaying differential Log_2_FC in obesity with p < 0.20 in either all EECs or L-type EECs. Using this criteria *RGS2, RGS4, RGS9, and RGS12* were identified as having physiologically relevant transcriptional alterations in obesity compared to lean (Fig. 1f) (Supplemental Table S2). In all-EECs *RGS12* displayed significantly increased expression in obesity (0.31 Log_2_FC, p = 0.0019), and a trend of increased expression in L-type EECs (Log_2_FC = 0.32, p = 0.20). A trend of increased *RGS9* expression was demonstrated in both all-EECs and L-type EECs in obesity compared to lean (Log_2_FC = 0.29, p = 0.19, Log_2_FC = 0.61, p = 0.10, respectively). In contrast, Both *RGS2*, and *RGS4* displayed significant decreased expression in all-EECs in obesity (−0.75 Log_2_FC, p = 0.043; −0.42 Log_2_FC, p = 0.012, respectively). Unlike *RGS9* and *RGS12*, alterations to *RGS2* and *RGS4* in all-EECs were not mirrored in L-type EECs.

### Intestinal RGS profile association with obesity status and BMI

**Fig. 2:**
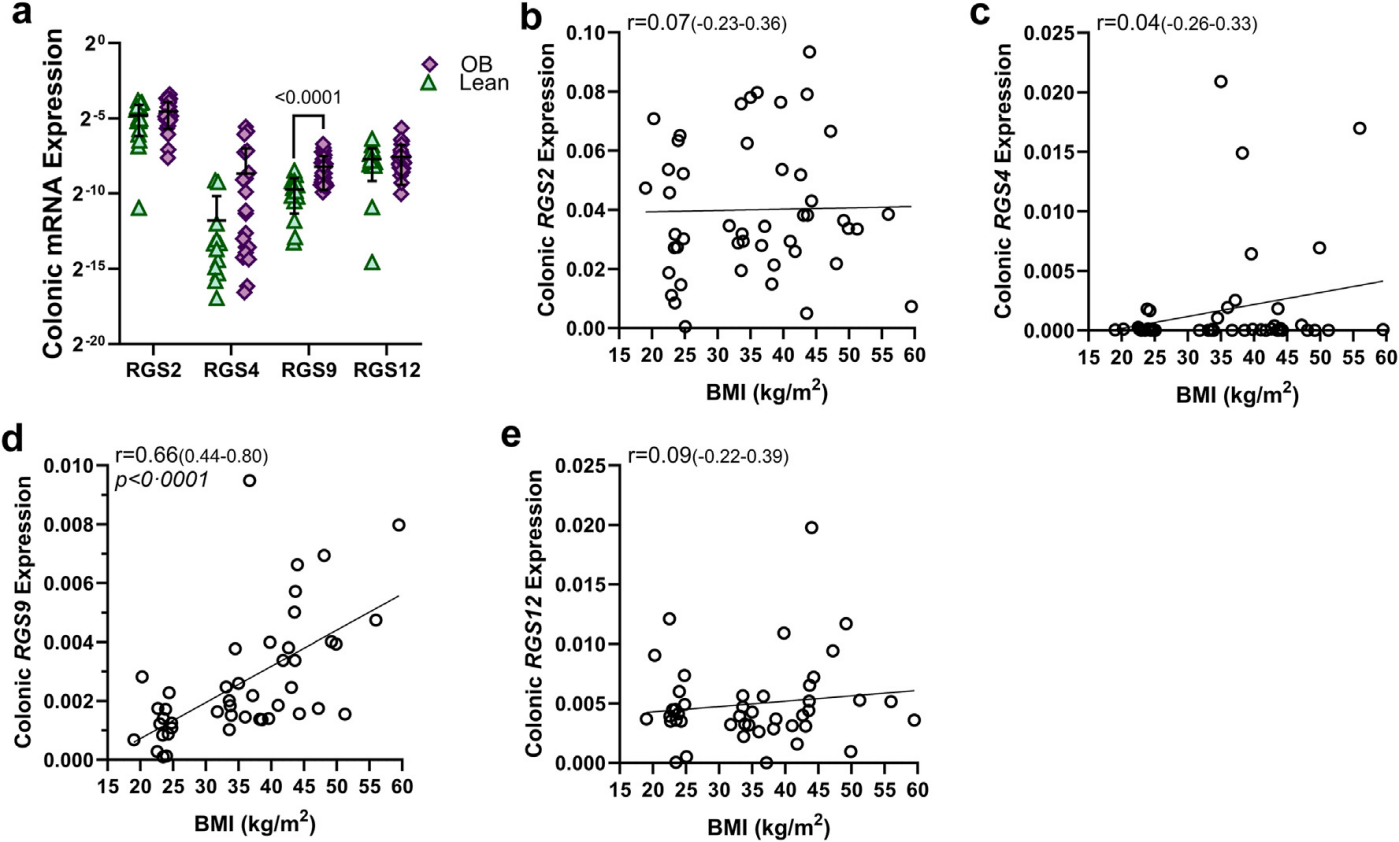
**Validation of the colonic enteroendocrine cell RGS profile and its intestinal transcriptomic alterations in obesity. a)** colonic *RGS* expression comparing lean (green triangle, n = 16 total cohort) and obesity (purple diamonds, n = 31 total cohort). Associations of BMI (kg/m^2^**)** with colonic mRNA expression of **b)***RGS2***c)***RGS4***d)***RGS9*, and **e)***RGS12*. Significance testing used a two-tailed unpaired Welch's t-test to compare between lean and obesity; data showing individual values within a group include a vertical error bar line, denoting SD, and a horizontal line denoting Mean value. The Spearman rank correlation analysis quantified the relationship between respective RGS expression and BMI. Spearman's correlation coefficient (r) is reported with 95% CI.

**Fig. 3:**
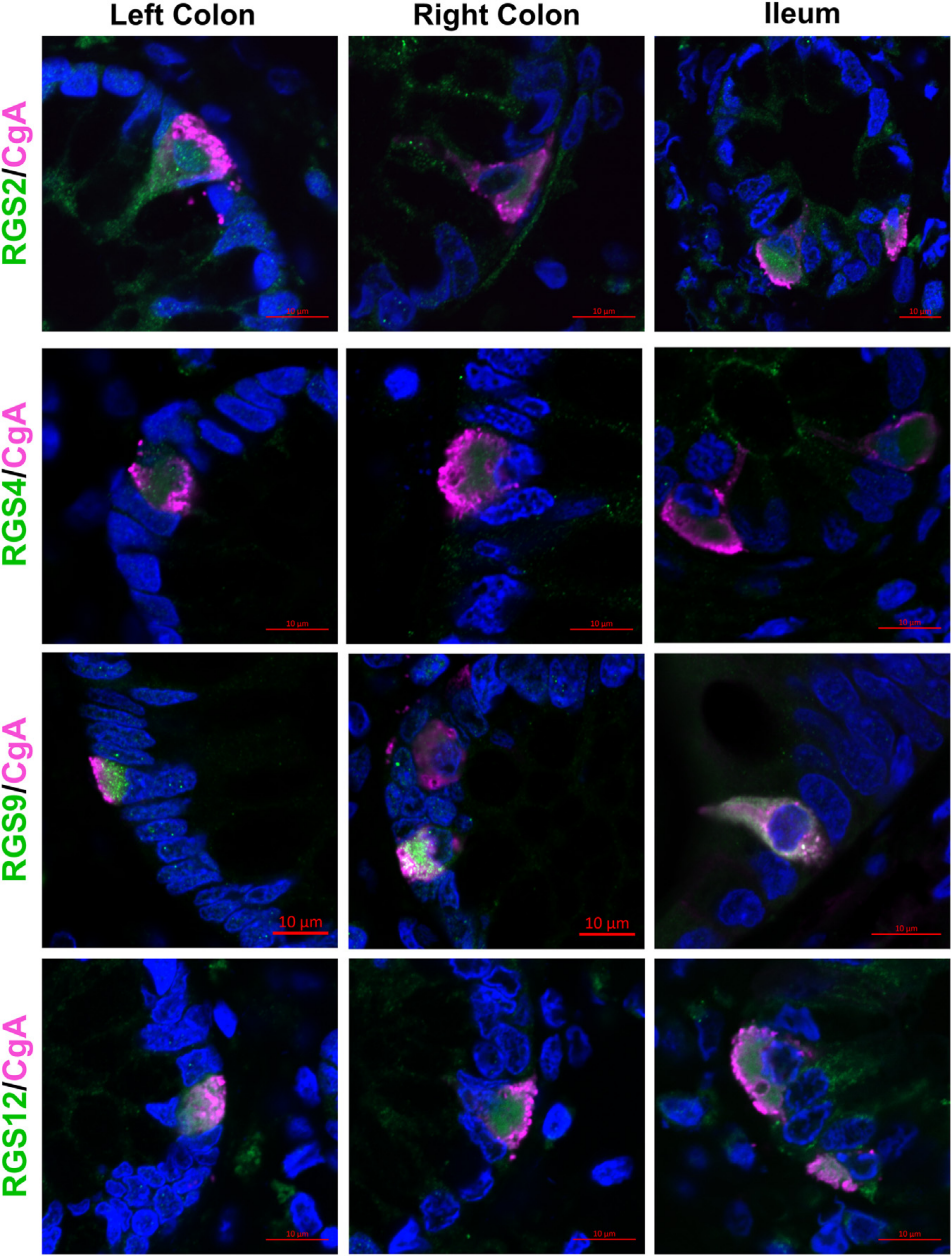
**Validation of RGS Expression in Human Intestinal Tissue.** Immunofluorescence (IF) staining of human mucosal colonic and ileal tissue sections demonstrating coexpression of RGS (green) with CgA (pink); red bar indicates 10 μm, nuclei stained with DAPI (Blue). 40x water Immersion objective.

In two validation cohorts we aimed to confirm the NGS findings of RGS expression in human intestine and further corroborate trends in differential RGS expression in obesity. In the first validation cohort (Table 1, “Single-cell RGS Family Tissue Validation”), we confirmed gut expression of *RGS* in both human ileum and colon in 15 participants. Expression of *RGS2*, *RGS11*, and *RGS14* was higher in the human ileum than colon, whereas expression levels of *RGS4*, *RGS9*, *RGS12*, and *RGS17* were similar (Supplemental Figure S2). In a second validation cohort (Table 1, “Colonic RGS Hormone Associations”) we sought to confirm our scRNAseq findings with respect to alterations in colonic *RGS2*, *RGS4*, *RGS9*, and *RGS12* in the context of obesity as both a categorical and quantitative variable in 16 lean healthy control patients and 31 patients with obesity. While the single-cell data set identified *RGS2*, *RGS4*, *RGS9*, and *RGS12* as having physiologically relevant transcriptional alterations within EECs in obesity, this finding was only validated for *RGS9* in the colon. Colonic *RGS9* demonstrated significant overexpression in obesity compared to lean (0.0033 ± 3.9e-004 vs. 0.0012 ± 2.1e-004, respectively; mean difference 0.002142 [95% CI 0.001250–0.003035], Log_2_FC = 1.50; p < 0.0001) (Fig. 2a). Protein expression of colonic RGS9 was additionally confirmed to be overexpressed in obesity (n = 10), compared to lean (n = 9) (1.23 ± 0.28 vs. 0.99 ± 0.11, respectively, mean difference 0.24 [95% CI 0.035–0.45], Log_2_FC = 0.30, p = 0.026) (Supplemental Figure S3). Colonic expression of *RGS2*, *RGS4*, and *RGS12* in obesity was not significantly different compared to lean. Assessment of RGS with respect to BMI also revealed a significant positive association with colonic *RGS9* (r = 0.66 (0.44–0.80); p < 0.0001); expression levels of *RGS2*, *RGS4*, and *RGS12* were not found to be associated with BMI (Fig. 2b–e). We further confirmed expression at the protein level in CgA-expressing human EECs in *RGS2*, *RGS4*, *RGS9*, and *RGS12* in human colon, and ileum (Fig. 3). Protein expression of RGS4 remained detectable, yet low in human intestine, consistent with validation mRNA expression data.

### Intestinal RGS association with GLP-1 and PYY expression and postprandial response

**Fig. 4:**
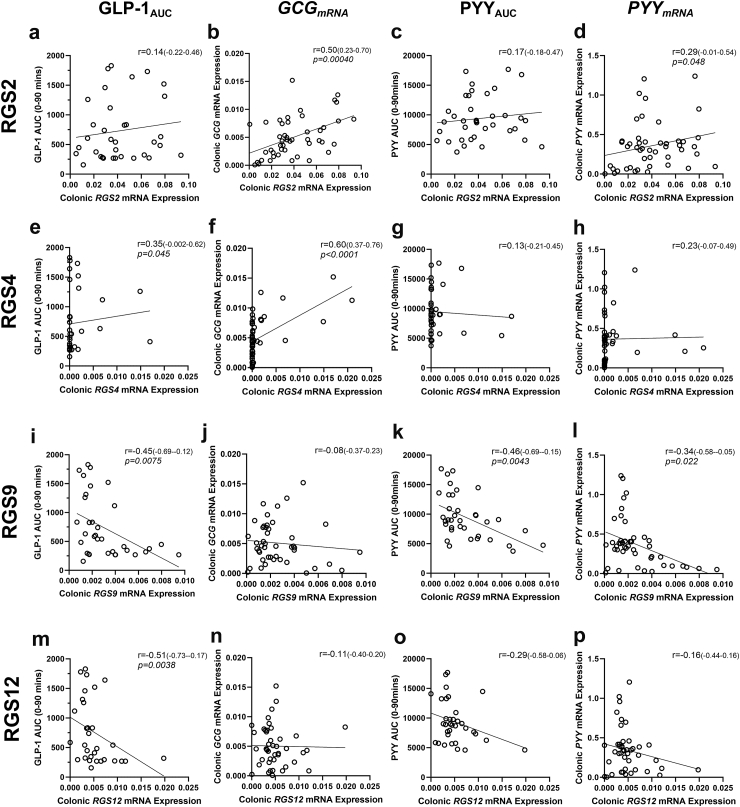
**Correlation of colonic RGS expression and satiety hormones.** mRNA Expression of colonic *RGS2* associations with area under the curve (AUC) for plasma concentrations of **a)** GLP-1, and **c)** PYY for time points 0–90 min postprandial (n = 34), and colonic expression of **b)***GCG* and **d)***PYY* mRNA (n = 47). mRNA Expression of colonic *RGS4* associations with AUC 0-90 mins for plasma concentrations of **e)** GLP-1, and **g)** PYY and colonic expression of **f)***GCG* and **h)***PYY* mRNA. mRNA Expression of colonic *RGS9* associations with AUC 0-90 mins for plasma concentrations of **i)** GLP-1, and **k)** PYY and colonic expression of **j)***GCG* and **l)***PYY* mRNA. mRNA Expression of colonic *RGS12* associations with AUC 0-90 mins for plasma concentrations of **m)** GLP-1, and **o)** PYY and colonic expression of **n)***GCG* and **p)***PYY* mRNA. Cohort of 47 participants with colonic biopsies, 34 of which also had postprandial plasma GLP-1 and PYY. Significance testing used the Spearman rank correlation test to quantify the relationship between the two tested variables in each panel. Spearman's correlation coefficient (r) is reported with 95% CI.

We next sought to determine the relationship between RGS and L-type EEC functionality in the context of GLP-1 and PYY mRNA expression and their respective postprandial blood levels in human subjects from the second validation cohort of 47 participants with colonic biopsies, 34 of which also had postprandial plasma GLP-1 and PYY. Colonic *RGS2* mRNA expression was positively associated with colonic *GCG* mRNA expression (r = 0.50 (0.23–0.70); p = 0.00040), and PYY mRNA expression (r = 0.29 (−0.01 to 0.54); p = 0.048) but not post prandial GLP-1 or PYY levels (Fig. 4a–d). Colonic *RGS4* mRNA expression was positively associated with both postprandial GLP-1 AUC_0–90 min_ (r = 0.35 (−0.002 to 0.62); p = 0.045) and colonic *GCG* mRNA expression (r = 0.60 (0.37–0.76); p < 0.0001), but not PYY colonic expression or postprandial levels (Fig. 4e–h). After ingestion of a meal, intestinal *RGS9* level was inversely associated with plasma post-prandial GLP-1 AUC_0–90 min_ (r = −0.45 (−0.69 to 0.12); p = 0.0075); but expression of *GCG* mRNA was not associated with *RGS9* mRNA (Fig. 4i–j). Similar to GLP-1, *RGS9* expression was also inversely associated with plasma post-prandial PYY AUC_0–90 min_ (r = −0.46 (−0.69 to 0.15); p = 0.0043), and further inversely associated colonic *PYY* mRNA expression (r = −0.34 (−0.58 to 0.05); p = 0.022) (Fig. 4k–l). A significant negative association was observed between *RGS1*2 mRNA expression and postprandial GLP-1 AUC_0–90 min_ (r = −0.51 (−0.73 to 0.17); p = 0.0038) (Fig. 4m). No associations were observed between *RGS12* and *GCG or PYY* mRNA expression or postprandial PYY blood levels (Fig. 4n–p).

### RGS inhibition modulates GPCR-mediated GLP-1 and release in human *ex vivo* cultures

**Fig. 5:**
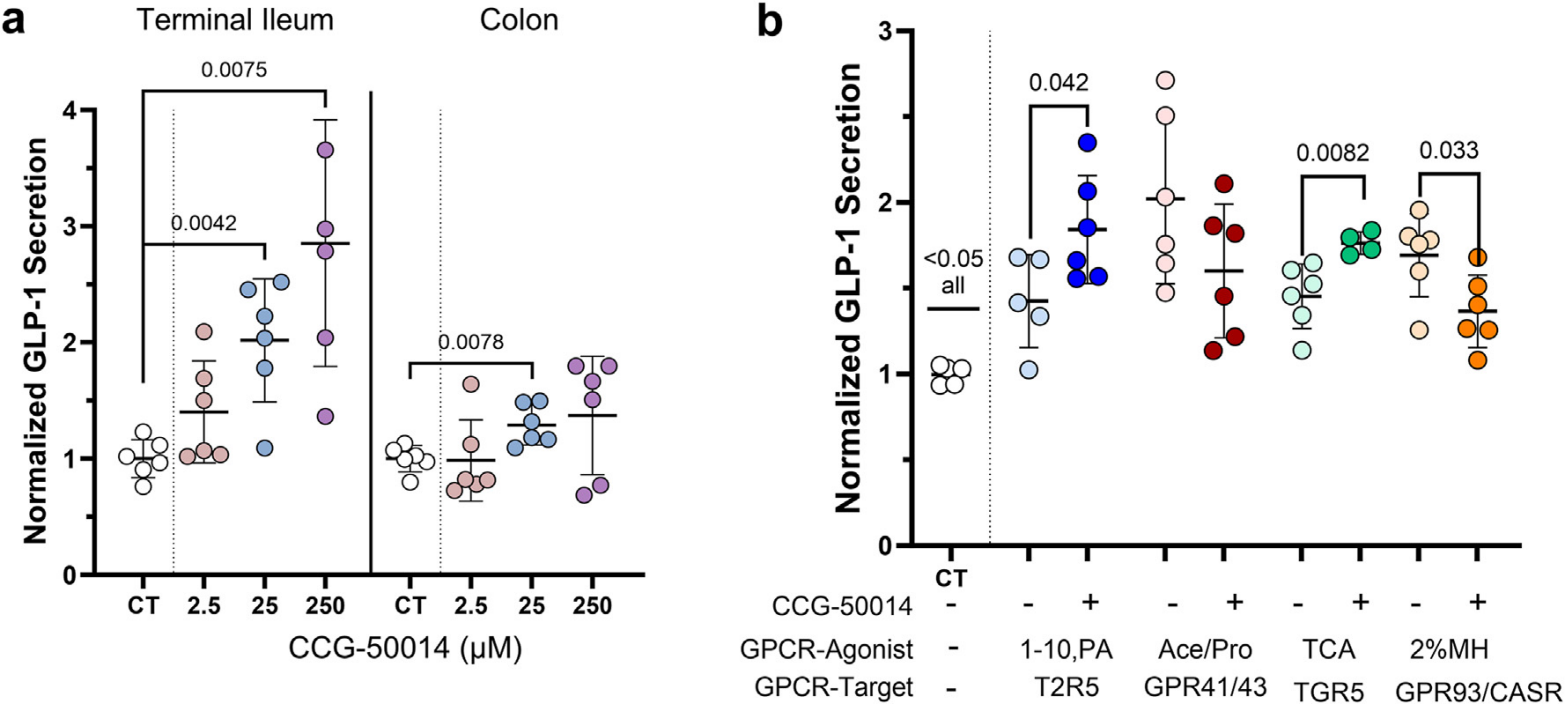
**Human gut RGS and functional relation to GPCR-mediated hormone secretion. a)** Primary cultures of human terminal ileum and colon were treated for 2 h with increasing doses of the nonspecific RGS inhibitor CCG-50014 and measured for secretion of GLP-1 into the media. **b)** Primary cultures of human terminal ileum were treated with known satiety hormone secretagogues (2% meat hydrolysate, 500 μM 1,10-Phenanthroline, 500 μM sodium acetate) in the presence or absence of 25 μM CCG-50014 and measured for secretion of GLP-1. Data expressed as fold-change to the respective controls. Data showing individual values within a group include a vertical error bar line, denoting SD, and a horizontal line denoting Mean value. Significance testing used a two-tailed unpaired Welch's t-test to compare between signified groups.

To investigate the consequences of RGS-alterations on GLP-1 hormone release, primary human intestinal monolayer cultures, originating from human colon and ileal tissue were exposed to a nonselective inhibitor of RGS activity, CCG-50014, at 3 doses (2.5, 25, 250 μM). Compared to control media, 2-h treatment of *ex vivo* tissue with 25 μM CCG-50014 increased the secretion of GLP-1 into the media approximately 2.02 ± 0.44-fold in terminal ileum (mean difference 1.02 [95% CI 0.46–1.57], p = 0.0042) and 1.29 ± 0.17-fold in colon (mean difference 0.29 [95% CI 0.098–0.48], p = 0.0078) (Fig. 5a). Additionally, treatment with 250 μM CCG-50014 in terminal ileum, but not colon potentiated secretion of GLP-1 compared to control (2.85 ± 1.06-fold change, mean difference 1.85 [95% CI 0.74–2.97], p = 0.0075).

We next explored how RGS inhibition would affect hormone response from EECs when used in conjunction with known GLP-1 secretagogue GPCR-agonists: bitter tastant 1,10-Phenanthroline (1,10-PA) targeting Taste 2 Receptor 5 (T2R5); short chain fatty acids acetate and propionate targeting GPR41/43; taurocholic acid (TCA) a bile acid targeting TGR5; and 2% meat hydrolysate (2% MH) targeting GPR93/CASR. Treatment of *ex vivo* human intestinal tissue with these agents potentiated the secretion of GLP-1 from human intestinal tissue compared with control (p < 0.05 all) (Fig. 5b). Secretion of GLP-1 was further augmented by the addition of CCG-50014 (25 μM) in conjunction with 1,10-PA and TCA (1.84 ± 0.31-fold, 1,10-PA + CCG-50014 vs. 1.42 ± 0.27-fold, 1,10-PA alone, mean difference 0.42 [95% CI 0.019–0.82], p = 0.042; 1.76 ± 0.07-fold TCA + CCG-50014 vs. 1.45 ± 0.19-fold, TCA alone, mean difference 0.31 [95% CI 0.11–0.51], p = 0.0082). Interestingly, the GLP-1 secretory effect of 2%MH was significantly dampened by the addition of the RGS inhibitor (1.37 ± 0.21-fold 2%MH + CCG-50014 vs. 1.69 ± 0.24-fold 2%MH alone, mean difference −0.33 [95% CI −0.62 to −0.033], p = 0.033), yet GLP-1 levels remained elevated compared to control (1.37 ± 0.21-fold 2%MH + CCG-50014 vs. 1.00 ± 0.06-fold, control, mean difference 0.37 [95% CI 0.15–0.59], p = 0.0070). Acetate/Propionate-stimulated GLP-1 secretion was not significantly altered by addition of RGS inhibitor.

### RGS9 overexpression modulates GPCR-mediated GLP-1 secretion *in vitro*

**Fig. 6:**
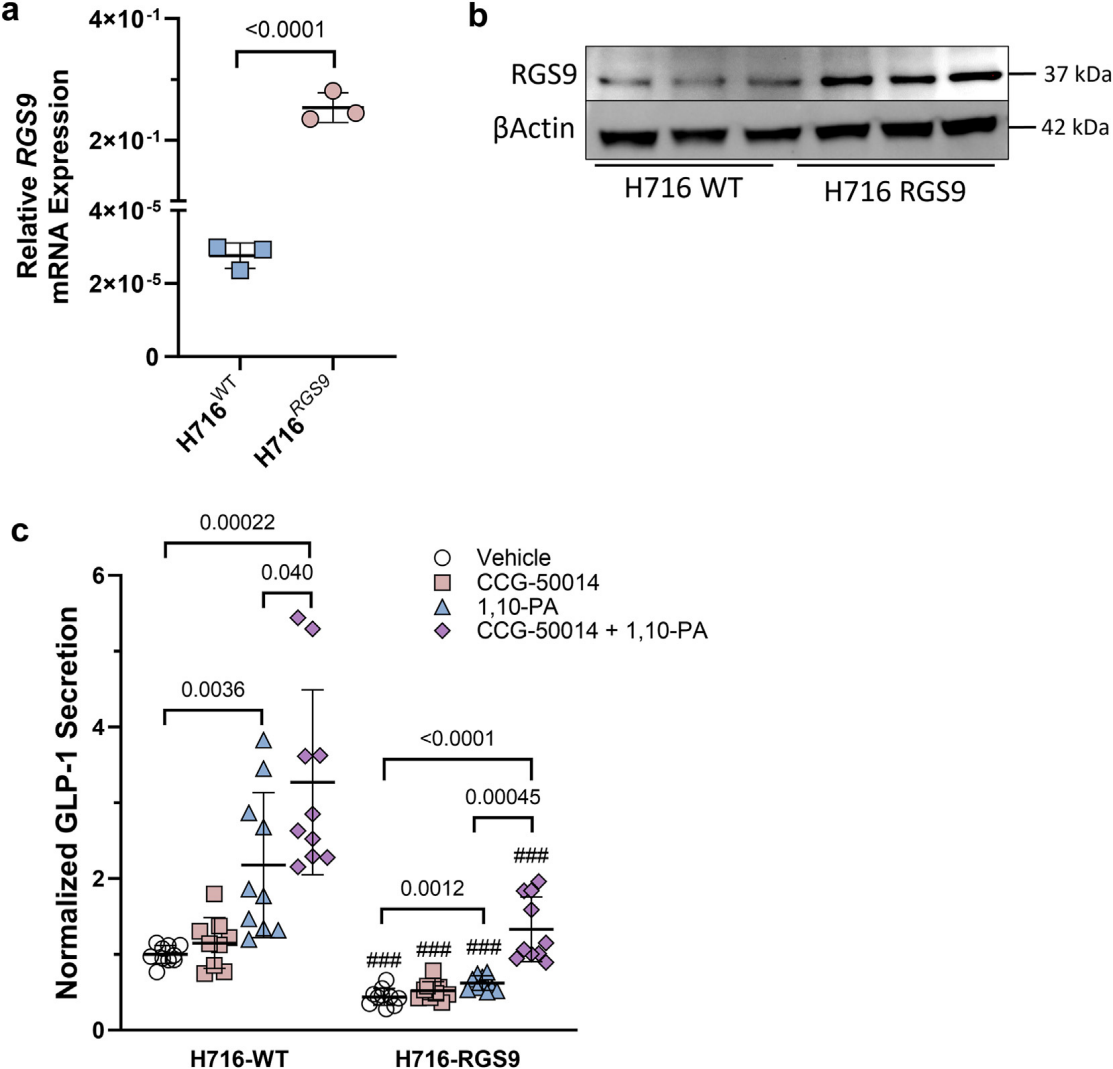
**NCI–H716 transgenic line overexpressing *RGS9* and GPCR-mediated hormone secretion**. **a)** Relative mRNA, measured by RT-qPCR and protein expression using **b)** Western blot and of *RGS9* in wild-type (H716^WT^) or *RGS9* overexpressing H716 cells (H716^RGS9^)**. c)** GLP-1 secretion from H716^WT^ and H716^RGS9^ cells treated for 2 h without and with CCG-50014 in response to 1,10 Phenanthroline (1 mM), compared to vehicle control (DPBS). Data expressed as normalized to protein content, and as fold-change to the wild-type control. Data showing individual values within a group include a vertical error bar line, denoting SD, and a horizontal line denoting Mean value. Significance testing used a two-tailed unpaired Welch's t-test to compare between signified groups among respective cells. Significance testing used a two-tailed unpaired Welch's t-test to compare GLP-1 between wild-type and H716^RGS9^ for respective treatments ###p < 0.001.

We further explored how RGS may influence GPCR-mediated secretion of GLP-1 from a human model of L-type EECs. First, we overexpressed *RGS9* in NCI–H716 cells (H716^*RGS9*^) (Fig. 6a and b), a nutrient-responsive human *in vitro* model of L-type EECs cells capable of secreting GLP-1 upon differentiation.^15^^,^^27^ Secretion of GLP-1 with vehicle control was significantly blunted in H716^*RGS9*^ with levels dampened by 56% compared to wild-type NCI–H716 (H716^WT^) (H716^WT^ vehicle, 1.00 ± 0.012 vs. H716^*RGS9*^ vehicle, 0.44 ± 0.11; mean difference −0.56 [95% CI −0.67 to −0.45], p < 0.0001) (Fig. 6c). Unlike *ex vivo* human intestinal cells, treatment with CCG-50014 (25 μM) alone was not sufficient to potentiate the secretion of GLP-1 from either the H716^WT^ or H716^*RGS9*^ compared to their respective vehicle controls. While both H716^WT^ and H716^*RGS9*^ appropriately responded to stimulation with bitter tastant GPCR-agonist 1,10-PA (1 mM) exposure with subsequent increased secretion of GLP-1 compared to their vehicle controls, H716^*RGS9*^ GLP-1 secretory response was blunted compared to H716^WT^ (1,10-PA vs. respective vehicle: H716^WT^ 2.18 ± 0.96-fold increase, mean difference 1.18 [95% CI 0.49–1.87] p = 0.0036; H716^*RGS9*^ 1.41 ± 0.22-fold increase, mean difference 0.18 [95% CI 0.083–0.28], p = 0.0012; ΔGLP-1 to 1,10-PA: H716^WT^ vs. H716^*RGS9*^ p = 0.033). Supplementation of CCG-50014 (25 μM) to 1,10-PA treatment further potentiated GLP-1 secretion an additional 1.5-fold in H716^*WT*^ (1,10-PA, 2.18 ± 0.96-fold increase vs. 1,10-PA + CCG-50014, 3.27 ± 1.22-fold increase; mean difference 1.09 [95% CI 0.059–2.13], p = 0.040), and compared to vehicle control GLP-1 secretion heighted 3.27-fold (1,10-PA + CCG-50014, 3.27 ± 1.22-fold increase vs. control, 1.00 ± 0.12; mean difference 2.27 [95% CI 1.40–3.15], p = 0.00022). Secretory GLP-1 response in H716^*RGS9*^ was also heightened with supplementation of CCG-50114 to the 1,10-PA treatment with GLP-1 increased 3.06-fold compared to respective vehicle control, (1,10-PA + CCG-50014, 1.33 ± 0.43 vs. vehicle, 0.44 ± 0.11; mean difference 0.89 [95% CI 0.58–1.20], p < 0.0001), and 2.1-fold compared to 1,10-PA treatment alone (1,10-PA + CCG-50014, 1.33 ± 0.43 vs. 1,10-PA, 0.62 ± 0.10; mean difference 0.71 [95% CI 0.40–1.02], p = 0.00045). However, GLP-1 levels for all treatments were significantly decreased in H716^*RGS9*^ compared to their respective treatments in H716^WT^ (p < 0.001, all).

## Discussion

The gut L-type EECs secrete satiety-inducing hormones GLP-1 and PYY after a meal in response to activation of their surface GPCRs by luminal components of nutrient digestion and microbial fermentation. The RGS family of GTPase-activating proteins (GAPs) are major negative regulators of GPCRs through their ability to rapidly induce GPCR signal termination. Prior to this study, RGS had yet to be directly investigated in EECs. In the present work, we identified and validated the expression of the RGS family of proteins in gut EECs and demonstrated their altered expression profile in obesity. Furthermore, RGS may modulate secretion of satiety hormones GLP-1 and PYY from EECs, and alterations to their expression may promote aberrant nutrient-mediated GPCR signaling.

The RGS family of proteins accelerate hydrolysis of the active GTP-bound Gα to the inactive GDP-bound Gα, effectively leading to the rapid termination of GPCR signaling.^16^ As these proteins represent a major mechanism for the negative regulation of GPCR signaling, we hypothesized that EECs, which rely heavily on chemosensory GPCRs, employ RGS proteins to coordinate the intricate balance between signaling activation and deactivation within the cell. Indeed, our transcriptomic profiling of human colonic enteroendocrine cells using scRNA-Seq and our analysis of a publicly available dataset profiling human jejunal enteroendocrine cells using RNAseq (GSE114853), demonstrated the expression of a unique set of RGS genes in human EECs of the colon and jejunum. A wide range of expression was displayed among the RGS family and appeared tissue- and cell type-specific. While expression of *RGS2* showed the highest overall expression in both colon and jejunum, it was not enriched in L-type EECs; furthermore, its colonic expression was limited to a minority of colonic L-type EECs. *RGS4, RGS7*, *RGS9, RGS11, and RGS12* were enriched in L-cells compared to non-EEC mucosal cells in both colon and jejunum, whereas among EECs, L-cell enrichment in both tissues was only observed in *RGS4*, *RGS9*, and *RGS14*.

Previous studies have associated tissue specific and global RGS alterations with human disease states including obesity.^17^^,^^18^ Here, we show in colonic EECs, obesity was associated with an altered RGS profile, with differential expression patterns in colonic *RGS2* (underexpression), *RGS4* (underexpression), *RGS9* (overexpression)*,* and *RGS12* (overexpression). Overexpression of whole colonic *RGS9* in obesity was further validated in whole human colonic mucosa and additionally associated with BMI.

We next aimed to characterize the functional role of RGS proteins in L-type EEC-dependent hormone secretion. The production and secretion of GLP-1 and PYY from human EECs *in vivo* can be indirectly studied by evaluating their fasting mRNA expression and concentrations in blood plasma in the fasting and postprandial state.^33^, ^34^, ^35^ Here we report that colonic *RGS2*, *RGS4*, *RGS9,* and *RGS12* may be of importance to human EEC functionality in regards to hormone production and or secretion, as evidenced by their associations with mRNA expression or postprandial plasma levels of GLP-1 and PYY.

Importantly, RGS9 expression displayed negative correlations with plasma concentrations of postprandial satiety hormones, in addition to its positive correlation with BMI. Overexpression of *RGS9* in an *in vitro* model of human L-type EECs was associated with blunted nutrient-stimulated GLP-1 secretion that was improved but not fully rescued upon administration of a non-selective RGS inhibitor in conjunction with a GLP-1 secretagogue. The non-selective inhibition of intestinal RGS potentiated the secretion of GLP-1 in *ex vivo* human intestinal tissue. Additionally, the GLP-1 secretory responses mediated by stimulation with GLP-1 secretagogue GPCR-agonists 1,10-PA (targeting bitter taste receptor T2R5) and TCA (targeting bile acid receptor TGR5) were further augmented with RGS inhibition in *ex vivo* human intestinal tissue. However, GLP-1 responses mediated through short chain fatty acid receptor activation with acetate/propionate were unaffected by RGS inhibition, and negatively affected with 2% MH-mediated stimulation of amino acid/peptide receptors. These differential GLP-1 secretory responses observed between the GLP-1 secretagogue GPCR-agonists are likely reflected by the selectivity of RGS proteins for Gα subunits of heterotrimeric G-proteins.^36^ A comprehensive understanding of the α-subunit linkage of relevant EEC nutrient sensing GPCRs coupled with current knowledge of RGS-Gα selectivity will be crucial for future studies aiming to dissect the dynamic relationship between RGS and their associated GPCRs in EECs and harness their potential to modulate GLP-1 secretion. While these data suggest an association between RGS, RGS9, and L cell hormone products, further studies are required to determine the exact mechanistic relationship between relevant RGS proteins and L cell functionality.

In the context of obesity the secretion of GI hormones may be impaired, as attenuated postprandial levels of GLP-1 and PYY have been characterized in some studies of obesity and negatively associated with appetite, length of postprandial satiety, and energy intake.^8^^,^^10^^,^^33^^,^^37^, ^38^, ^39^, ^40^ In the present study we show intestinal RGS expression is altered in obesity and associated with postprandial GLP-1 and PYY. However, In our cohorts fasting and postprandial GLP-1 and PYY were not altered in obesity; therefore, it is unlikely overexpression of RGS9 represents a universal pathophysiological process in obesity. However, phenotypic subgroups of obesity characterized by abnormal postprandial satiety hormone responses have been reported, and investigating RGS function in such patients could illuminate a pathophysiologic process.^41^

Many pharmacological attempts have been made to simulate the physiological effects of L-cell satiety hormone peptides GLP-1 and PYY as a treatment for obesity,^1^^,^^5^ and the findings presented in the current manuscript indicate that consideration of EEC-specific RGS may present new beneficial applications to therapeutic strategies in GPCR-based drug discovery for obesity. The RGS family, specifically *RGS9*, may serve as novel targets for the modulation of satiety hormone secretion in obesity. Additionally, our study indicates that even in the presence of a potent small molecule GLP-1/PYY secretagogue, there still may exist an intracellular signaling blockade, mediated by altered RGS expression, preventing full secretion potential. Thus, a powerful and potentially necessary pharmacological approach to stimulating maximal endogenous secretion of these satiety hormones may include the combination of GPCR agonists coupled with EEC-specific RGS inhibitors.

It is important to note some limitations of our investigation. First, transcriptomic profiling was completed in human jejunal and colonic EECs, while validation and further studies included human ileal and colonic tissue. Controversy remains regarding the significance of colonic EECs and GLP-1 from colonic origins on food intake, and potential differences in nutrient-stimulated responses between L-type EECs originating from colon and small intestine.^42^ However, EECs are distributed throughout the GI tract, the concentrations of GLP-1 and PYY are similar in the ileum and colon,^43^^,^^44^ and the current literature describes colonic EECs, similar to their ileal and jejunal counterparts, capable of influencing postprandial plasma hormone responses.^45^^,^^46^ Second, the initial discovery cohort studied EEC-specific RGS, whereas the validation cohorts utilized whole tissue and therefore were not specific to EECs. Further studies in isolated populations of EECs will be important. Third, our functional *ex vivo* and *in vitro* studies utilized a nonselective RGS inhibitor, therefore we cannot fully conclude a role for specific RGS proteins. Finally, the experimental design of our studies did not control for confounding effects, which may have biased the study results. Detailed mechanistic studies will be important to further elucidate the role of RGS proteins in enteroendocrine cell physiology.

This study introduces RGS proteins as likely regulators of satiety hormone secretion from EECs. Alterations in gut RGS levels may play a role in EEC pathophysiological processes and could represent novel therapeutic targets in obesity.

## Contributors

All authors read and approved the final version of the manuscript. Individual contributions to the manuscript were: Conceptualization, AM and AA; Methodology, AM, AT, VS, YL, and AA; Validation, AM, AT, NM, FFJ and MRS; Formal Analysis, AM, YL, YL, and AA; Investigation, AM, MRS, AT, YL, YL, FFJ, DGI, GC, FR, VS, DG, CL and AA; Writing—Original Draft: AM and AA. Writing—Review & Editing, AM, AT, MRS, NM, FFJ, DGI, GC, FR, YL, YL, VS, CL, DH, NL and AA; Visualization, AM, YL, MRS, and AA; Supervision, YL, NL and AA; Project Administration, AM and AA; Funding Acquisition, AM and AA.

## Data sharing statement

Data collected for the study, including individual de-identified participant data, as well as the study protocol and informed consent will be available to interested parties with publication after signing of a data access agreement. Data may be requested by contacting Dr. Andres Acosta M.D, Ph.D., at acosta.andres@mayo.edu. Partial sequencing data generated during this study are included in this published article and its supplemental information files. Full data is publicly available from the Gene Expression Omnibus (GEO) at the National Center for Biotechnology Information (NCBI) repository under accession number: GSE154405.

## Declaration of interests

AA is a stockholder in Gila Therapeutics, Phenomix Sciences. AA provides consulting services for Rhythm Pharmaceuticals, General Mills, Amgen, RareStone, and Bausch Health. DH provides consulting services for Novo Nordisk and receives presentation compensation from the Obesity Medicine Association and The Menopause Society.

## Figures and Tables

**WB BACT Original.png:**
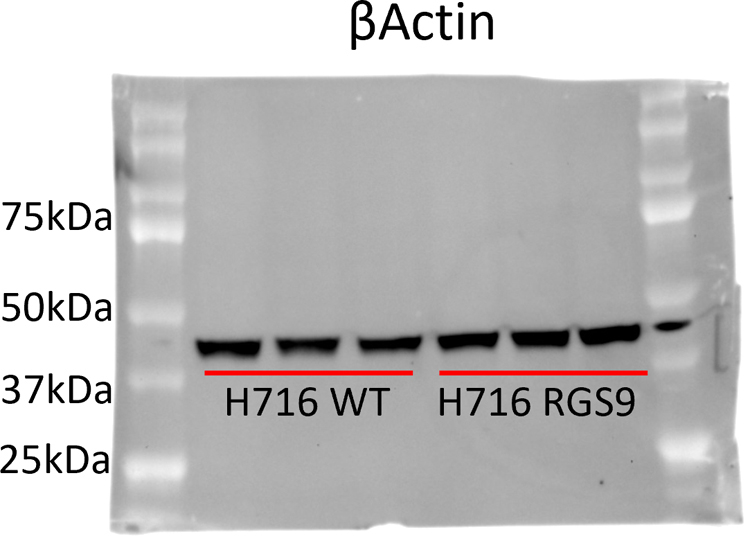


**WB RGS9 Original.png:**