# Supplementary material for: Regulator of G-protein signaling expression in human intestinal enteroendocrine cells and potential role in satiety hormone secretion in health and obesity
Source: eBioMedicine. 2024 Aug 13;107:105283. doi: 10.1016/j.ebiom.2024.105283 (PMC11367526; doi:10.1016/j.ebiom.2024.105283)
Supplement: Supplementary Material [file mmc1.docx]

**SUPPLEMENTARY MATERIALS**

**METHODS**

**Participants, Tissue Collection and Hormone Testing**

The study was approved by the Mayo Clinic Institutional Review Board, and all participants gave written informed consent following thorough explanation of the study details. Women of childbearing potential had a negative pregnancy test within 48 hours prior to testing. We included men and women, age 18-65, having a stable weight for the previous 3 months. Sex/gender was self-reported by study participants. We excluded patients with recent use of weight loss medications (<6 months), history of abdominal GI surgery other than appendectomy, pregnancy, uncontrolled systemic disease, or medications that might interfere with motility, appetite, or absorption.

Studies were performed at the Mayo Clinic Clinical Research Trials Unit (CRTU) after an 8-hour fasting period. Endoscopic mucosal tissue was collected from a total of 61 participants (lean n=19; obesity n=42) after receiving a tap water enema during a flexible sigmoidoscopy, or during routine colonoscopy with ileal intubation **(Table 1, “Complete Cohort”).** During the procedure, 8-16 mucosal biopsies were obtained from the colon and/or ileum. Tissue was then processed for appropriate downstream applications, as described below.

For determination of plasma postprandial GLP-1 and PYY, participants received a mixture of 63g glucose in 240 ml of skim milk and a meal with 2 scrambled eggs, 50g of Canadian Bacon and one slice of bread (~560 Kcal: 43% carbohydrate, 18% protein, and 40% fat). Plasma samples were collected fasting and postprandial at 15, 45 and 90 minutes for measurement of GLP-1 (Cat#GLP1T-36HK, Millipore Sigma), and PYY (Cat#PYYT-66HK, Millipore Sigma).

**Single-Cell RNA-Sequencing and Analysis**

*Tissue Collection and Cryopreservation*

Mucosal biopsies originating from the sigmoid colon were collected, cryopreserved, and prepared for scRNA-Seq as previously described.(1) Briefly, after an 8 hour fasting period participants underwent an unsedated flexible sigmoidoscopy subsequent to receiving a tap water enema. 13 mucosal biopsies were obtained from the sigmoid colon using a single-use Radial JawTM 4 Jumbo Biopsy Forceps (M00513361, Boston Scientific). Biopsies were placed in 1ml chilled HypoThermosol FRS preservation solution (101102, BioLife Solutions), and transferred on ice to the processing laboratory within 15 minutes of collection. Biopsies were washed with cold sterile PBS, placed in a cryogenic vial (5000-1020, Thermo Scientific) containing 1ml sterile CryoStor® CS10 cryopreservation media (07930, StemCell Technologies), and incubated for 10 minutes at room temperature. Cryogenic vials containing biopsies were placed in a Mr. Frosty Freezing Container (51000001, Thermo Scientific) and stored at -80°C. After 24-hours, samples were transferred to a dewar containing liquid nitrogen, and stored until further use.

*FACS-Isolation*

On the day of the experiment, cryopreserved biopsies were rapidly thawed in a 37°C water bath for 5 minutes. The cryopreservation media was removed from the sample and replaced with 1ml of pre-warmed RPMI medium (25-506, Genesee Scientific) supplemented with 10% FBS (v/v) for 15 minutes at room temperature. Biopsies were washed with cold sterile-PBS and transferred to a low-binding RNase-free microcentrifuge tube (AM12450, ThermoFisher Scientific). Biopsies were minced with surgical scissors and washed twice with Hanks' Balanced Salt Solution formulated without calcium or magnesium(HBSS –Ca2+, –Mg2+; 14170112, Gibco), and dissociated by enzymatic collagenase digestion. The resulting single cell suspensions were stained with the Membrane Permeability Dead Cell Apoptosis Kit (V35123, Invitrogen) with PO-PRO-1 (early apoptosis marker) and 7-AAD (viability), and sorted using a BD FACSAria II cell sorter. In order to set appropriate gates for sorting, 50,000 events for each unstained, and FMO controls were run. Cell yield and sample composition was further assessed using FlowJo (FlowJo v10.6, FlowJo, LLC). All samples were subjected to the same gating strategy to isolate single, live, non-apoptotic cells. Cells were isolated into 2ml of PBS containing 2% (w/v) bovine serum albumin (BSA). Cells were then washed with 1ml sterile RNase free PBS and filtered with a 30-μm cell strainer.

*scRNA-Seq and Data Analysis*

We performed all steps following the Chromium 10X Genomics single cell RNA-Sequencing (scRNA-Seq) platform., with a targeted cell capture of 10,000 single cells. We used the Chromium Single Cell 3′ Library & Gel Bead Kit v2 (10X Genomics). In short, all samples and reagents were prepared and loaded into the chip. Then, we ran the Chromium Controller for droplet generation. Reverse transcription was conducted in the droplets. We recovered cDNA through demulsification and bead purification. Pre-amplified cDNA was further subjected to library preparation. Libraries were sequenced on an Illumina Hiseq 4000 for 100 paired-end runs at 1 sample over 2 lanes.

We used 10X Genomics Cellranger Single Cell Software Suite (v3.0.0) to generate FASTQ files, perform alignment to hg38 reference genome, filtering, barcode counting and UMI counting. For subsequent clustering (k-means) and downstream analysis, we followed the integrated analysis workflow in the Seurat package (v3.1) (https://satijalab.org/seurat/v3.1/integration.html). Genes that are expressed in fewer than 3 cells, cells that expressed fewer than 200 genes and >40% mitochondria genes were excluded for downstream analysis in each sample. Each dataset was normalized using log normalization and scaled for each gene across all cells. All datasets were integrated, scaled, and clustered on the low-dimensional space. Resolution parameter for Seurat was set to 0.3 for all data integrations. Enriched gene markers in each cluster conserved across two conditions were identified with fold change larger than 2, adjusted p-value smaller than 0.05 in both conditions. All clustering and statistical analysis was performed in R (v 3.5.2).

**Analysis of GSE114853 RNA-Sequencing Database**

*Data Acquisition*

The “Human enteroendocrine cell transcriptomic profiling” GSE114853 RNAseq dataset and their associated information were obtained from the NCBI Gene Expression Omnibus (GEO). Methodology for the comparative transcriptomic study is fully described as previously published (2). Briefly, transcriptomic profiling of 3 cell populations of human jejunum in 11 participants was completed by bulk RNAseq using Illumina HiSeq 4000. Cell populations included FACS-purified populations of human jejunal enteroendocrine cells: L-type jejunal EECs (GLP1+/CHGA+/SCG2+), non-L-type jejunal EECs (GLP1-/CHGA+/SCG2+), and the third population contained non-EEC jejunal cells (GLP1-/CHGA-/SCG2-).

**Enteroendocrine Physiology Validation Studies**

*Participants*

We interrogated the gut hormone expression profile of 47 participants within our studies for either, mucosal mRNA and protein expression, plasma hormone levels, or both (**Table 1, “Colonic RGS Hormone Associations”**). In a cohort of 15 participants both colonic and ileal biopsies were collected for validation of RGS expression **(Table 1, “Validation: Single-cell RGS family tissue validation”)**. Colonic and ileal mucosal biopsies, as well as postprandial plasma samples were collected as described above. Colonic and ileal mucosal biopsies were either immediately cryopreserved, placed in RNA*later* (Ambion) for subsequent RT-qPCR, or fixed in 10% NBF for immunofluorescence studies.

*Traditional RT-qPCR*

Total RNA was extracted from RNAlater preserved colonic biopsies using the RNeasy Plus Micro Kit (Cat#74034, Qiagen), and resulting RNA concentration and purity (A260/A280) was determined using a NanoDrop Spectrophotometer (Cat#ND-2000, ThermoFisher). The RNA was reverse transcribed into 0.5µg cDNA in 20µl reactions using the AffinityScript QPCR cDNA Synthesis Kit (Cat#600559, Agilent Technologies) according to the manufacturer’s instructions. The cDNA (0.1µg) was amplified by real-time quantitative PCR using gene-specific primers (250 nM final concentration) and performed in the LightCycler 480 II System (Roche Life Sciences) using SYBR green (Cat#172-5270, Bio-Rad) detection. All primers were designed using the NCBI Primer BLAST tool, and manufactured by Integrated DNA Technologies (**Supplemental Table 1**). Eukaryotic Elongation Factor 2 (EEF2) was used as an endogenous control to normalize each sample(3, 4). Gene expression was calculated using the 2-ΔΔCt method and presented as normalized gene expression to *EEF2* expression for each sample.

*RT^2^ PCR Array*

Hit validation from the scRNA-Seq study was conducted using a custom 384-well plate RT^2^ Profiler PCR Array (Cat#CLAH39276, Qiagen), generated with specific RGS-family genes. Total RNA was extracted from endoscopic biopsies obtained from human colon and ileum of 15 participants (Lean, n=6; Obesity, n=9) as described above. cDNA (400ng, 20µl reactions) was synthesized using the RT2 First Strand Kit (CAT#330404, Qiagen) according to the instructions of the manufacturer. The cDNA was amplified by real-time quantitative PCR using the LightCycler 480 II System (Roche Life Sciences) using SYBR green detection according to the instructions of the RT² SYBR Green qPCR Mastermix (CAT#330503, Qiagen). Beta-Actin (ACTB) was used as an endogenous control to normalize each sample Gene expression was calculated using the 2-ΔΔCt method, and presented as normalized gene expression for each sample, and fold-change compared to the control (lean) group. Appropriate no template, and no RT controls were run on each plate.

*Immunofluorescence*

Human mucosal colonic biopsies were fixed in 10% NBF overnight. The fixed tissue was incubated in 70% ethanol for 48 hours, and stored in sterile PBS at 4°C until embedding took place. Tissue was embedded in paraffin, and slides containing 5µm FFPE sections were prepared. The sections were deparaffinized and rehydrated through a graded alcohol series, followed by antigen unmasking where sections were boiled in Antigen Unmasking Solution (Cat#H-3300-250, Vector Laboratories) in a microwave for 20 minutes, and cooled for an additional 30 minutes. Tissue was permeabilized with 0.1% Triton X-100 in PBS for 10 minutes at room temperature, and blocked with 10% FBS, 1% BSA, 0.1% Tween-20 in PBS for 1 hour at room temperature. Sections were probed with primary antibodies specific for RGS2 (rabbit polyclonal, Cat# PA5-102486, Invitrogen, 1:300), RGS4 (rabbit polyclonal, Cat# PA5-22332, Invitrogen, 1:500), RGS9 (rabbit monoclonal, Cat#ab108975, Abcam, 1:100), RGS12 (rabbit polyclonal, Cat# PA5-65886, Invitrogen, 0.5µg/ml), and CgA (mouse monoclonal IgG_1_, Cat#MA5-13096, Invitrogen, 1:200). Fluorochrome conjugated secondary antibodies (Goat anti-Rabbit IgG Alexa Fluor Plus 647, Cat#A32733, Invitrogen, 1:600; Donkey anti-Rabbit IgG Alexa Fluor 488, Cat# A21206, Thermo fisher Scientific, 1:600, Goat anti-Mouse IgG Alexa Fluor 647, Cat# A32728, Invitrogen, 1:600, Goat anti-Mouse IgG Alexa Fluor 633, Cat#A-21050, Invitrogen,1:600) were used to detect respective primary antibodies. Slides were mounted with ProLong™ Gold Antifade Mountant with DAPI (Cat#P3693, Life Technologies). Images were taken on a Confocal Microscope (LSM 980 Axio Observer), and probes were excited using 405, 488, and 633 nanometer laser lines. Resulting images were analyzed with the ZEN software (ZEN 2.1, Zeiss).

*Semiquantitative RGS9 Protein Quantification*

Protein level of RGS9 was quantified in colonic biopsies of lean, and participants with obesity using semiquantitative immunohistochemistry. Preparation of FFPE slide sections from human colonic biopsy tissue, and subsequent IHC/IF Staining for RGS9 was completed as described above in “*Immunofluorescence*”. A negative, and a no primary antibody control, where primary antibody incubation was omitted, was used as negative controls to control for background fluorescence. Immunofluorescence analysis was performed in a blinded fashion by numerically coding for each participant in a random manner. The fluorescence staining was visualized, and probes were excited using the 488 nanometer laser lines. Images for each protein analysis were obtained using the same laser intensity and acquisition settings. Images were visualized for data analysis in the Zen Software, and quantification of the fluorescence intensity of the staining was performed by analysis of 3 random fields per participant using the “Histo” function of the ZEN software whereby the arithmetic mean intensity was multiplied by the count for each field after incorporation a lower threshold to optimize for background noise.

*Generation of Primary Cultures from Human Intestinal Biopsies*

Primary culture of intestinal monolayers, originating from human colon and ileum were generated using previously reported methodologies(5, 6). Briefly, thawed biopsies were rinsed with PBS, minced with surgical scissors, and digested with 10ml collagenase XI (1.25 mg/ml) in PBS at 37°C for 30 minutes with gentle rotation. Supernatant fractions from each digest were centrifuged twice at 100 g for 5 minutes to pellet crypts, and resuspended in 8 ml culture medium (DMEM Glutamax, 4.5g/L glucose, Cat#10566024, Gibco; supplemented with 10% (v/v) FBS, 100 units/ml penicillin and 0.1 mg/ml streptomycin). Aliquots (100 μl) were plated into 96-well plates coated with 4mg/ml Matrigel, and primary cultures were incubated for 4-hours at 37°C in 5% CO2.

*NCI-H716 General Cell Maintenance*

The Human L cell line NCI-H716 was obtained from the American Type Culture

Collection (CCL-251, ATCC) and maintained at a density of ~500,000 cells/ml in suspension culture in T25 suspension flasks (5669-0195, USA Scientific) containing RPMI medium (25-506, GenClone) supplemented with 10% FBS, 100 IU/ml penicillin, and 100 μg/ml streptomycin. Cells were kept at 37°C, 5% CO_2_, until 85% confluent, then split 1:5 and transferred to fresh T25 flasks.

*Transfection of NCI-H716 cells and generation of stable expressing RGS9 NCI-H716 cells*

NCI-H716 cells were cultured in RPMI-1640 medium with 10 % (v/v) FBS and grown at 37 °C in 5 % CO_2_. A RGS9 construct containing human RGS9-2 (accession number NM_001081955.3) in pcDNA 3.1 expression vector was purchased form GenScript (Clone ID: OHu05606). Transfection of NCI-H716 cells was performed using Lipofectamine 3000 (Invitrogen) following manufacturer’s instructions. Transfected cells were selected by selection media containing Geneticin G418 (100 μg/ml, Gibco). Individual colonies were isolated after 2 weeks of antibiotic selection and then were passaged continuously in a selection medium.

*Western Blot*

Cells were lysed by RIPA buffer containing protease inhibitors (Cat#A32963, ThermoFisher) after various experimental treatments, homogenized by sonication and centrifuged at 13,000 rpm for 10 min at 4°C to remove insoluble debris. The cell lysates were boiled in SDS sample buffer and then subjected to SDS-PAGE. After electrophoresis, proteins were transferred to PVDF membrane. Non-specific binding was blocked by incubation in 5%(w/v) BSA in PBS containing 0.1 % tween 20 (PBS-T). Membranes were blotted with anti-RGS9 antibodies in 5(w/v)% BSA in PBS-T at 4°C overnight. The membranes were then washed with PBST and incubated for 2 hours at room temperature with HRP conjugated anti-rabbit IgG (Cell signaling technology, MA) in blocking buffer. After washing with PBST, the blots were detected with an ECL kit and imaged using a ChemiDoc Imaging System (Bio-Rad).

*NCI-H716 Enteroendocrine Differentiation*

NCI-H716 cells were split into 96-well plates pre-coated with 150 µl per well 4 mg/ml matrigel (354234, Corning), at a density of 100,000 cells per well in 200µl media containing high glucose DMEM (11965092, Gibco) supplemented with 10%(v/v) FBS, 100 IU/ml penicillin, and 100 μg/ml streptomycin. Matrigel was diluted to a working concentration with serum-free DMEM. Cells were then kept at 37°C, 5% CO_2_ for 48 hours to allow for enteroendocrine differentiation.

*RGS Inhibition Cell Culture Experiments*

Primary human intestinal cultures were washed with DPBS and exposed to treatments (200µl, n=6 wells/treatment) for 2-hours. Treatments included: varying doses of RGS-inhibitor CCG-50014 (Cat#10802, Caymen Chemical) alone, or stimulating GPCR-agonists: meat hydrosylate (2% w/v; Cat#70174, Sigma), 1,10-Phenanthroline (500µM; Cat#131377, Sigma), Sodium Acetate/Propionate (500µM each; Cat#S8750, P5436, Sigma), taurocholic acid (500µM; CAT#T4009, Sigma). All treatments were prepared in DPBS-0.5% BSA (w/v) control media. For this challenge, we determined a CCG-50014 inhibitor dosage range based on dosages previously described to effectively attenuate RGS activity in RGS14 and RGS10 proteins, members of the R12 RGS protein family, most closely related to the R7 RGS family, which includes RGS9.(7) A control group was exposed only to PBS-0.5% BSA. Cells were incubated at 37°C for 2 hours, after which media was collected and centrifuged (5 minutes, 1,000 x g, 4°C) to pellet any cells, and the supernatant was then frozen at -20°C for subsequent ELISA analysis. Cell viability was measured using the Trypan Blue exclusion test. The active GLP-1 concentration of the media supernatants was determined by ELISA (Cat#EZGLPHS-35K, EMD Millipore Sigma) according to the instructions of the manufacturer. The active GLP-1 levels from each sample were then expressed as fold to their respective controls.

Differentiated NCI-H716 cells were thoroughly washed with DPBS and treated for 2 h with DPBS containing 0.5% BSA and either 1) vehicle (1:1000 DMSO and 1:1000 EtOH), CCG-50014 (25 µM), 1,10-phenanthroline (1 mM), or CCG-50014 (25 µM) and 1,10-phenanthroline (1 mM). Samples were processed as above and GLP-1 was measured in supernatant.

**Ethics Statement**

This study was approved by The Mayo Clinic Institutional Review Board (IRB), Rochester, MN, United States (Protocol numbers: 17-009999, 17-009678, 16-007060,16-008664). All authors had access to the full data, reviewed and revised the manuscript, and gave approval to submit the manuscript for publication.

**Statistics**

Data are expressed as mean ± SD unless otherwise stated. Data for differential gene expression in the RNA sequencing datasets were analyzed using the default Seurat package settings, based on the non-parametric Wilcoxon rank sum test. Graphical data from the RNA sequencing datasets are visualized as bar charts, where top horizontal line of bar represents average expression or proportion, and further denoted with exact numbers above individual bars. The nonparametric Spearman correlation analysis was completed to measure the associations between RGS expression and BMI, human plasma PYY AUC and GLP-1 AUC, and fasting colonic *PYY* and *GCG* mRNA expression. Data for nonparametric correlations are expressed as Spearman correlation coefficients (r) and 95% confidence interval (CI), with accompanying regression lines fitted with ordinary least squares. Significance testing comparing groups for relative RGS levels, normalized GLP-1 cell secretion from human intestinal cultures *ex vivo* and NCI-H716 cells *in vitro*, used a two-tailed unpaired Welch’s t-test assuming unequal variances, unless otherwise stated. The distribution of datasets were tested for normality using the Shapiro-Wilks test and through the generation of quantile-quantile (Q-Q) plots. Graphical representations of data showing individual values within a group include a vertical error bar line, denoting SD, and a horizontal line denoting mean value. Data were analyzed with the JMP Pro (Version14, JMP Statistical Discovery, LLC) statistical software. Visualization of data in the form of figures was completed using GraphPad Prism (Version 9.3.0, GraphPad Software, LLC).

**Role of the Funding Source**

The funding sources had no role in the study design; in the collection, analysis, and interpretation of data; in the writing of the report; and in the decision to submit the paper for publication.

**SUPPLEMENTARY FIGURES**


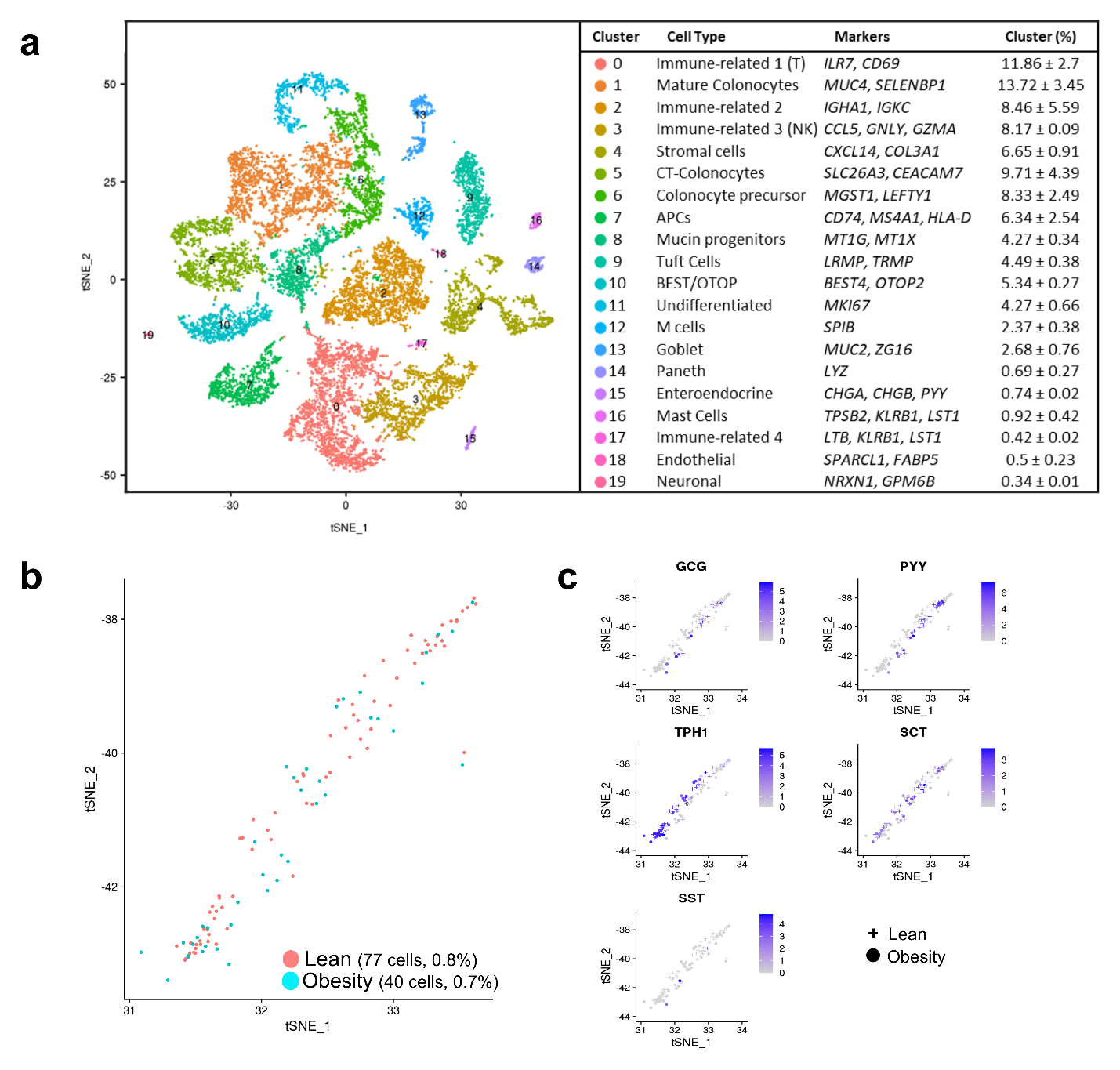


**Supplemental Figure 1.**  Single-cell RNA-Seq profiling of human colonic mucosa. **a)** Representative t-SNE plot of single-cell RNA-seq profiles of human intestinal mucosal cells, colored by cluster number and identity, listed in numerical order of cluster assignment, annotated by cluster identity, determined by gene markers for subtypes, and the average percentage (Mean ± SD) of cluster contribution to total cell population profiled. T-SNE plots showing zoomed view of the EEC cluster composition (modified from *McRae et al., 2022*) (1) **b)** demonstrating number of cells profiled for lean (pink dots) and patients with obesity (blue dots), and **c)** depicting expression levels of EEC-subtype gene markers within the EEC cluster, for lean (plus) and patients with obesity (circle), where shading intensity represents relative fold-increase in conserved expression.

**
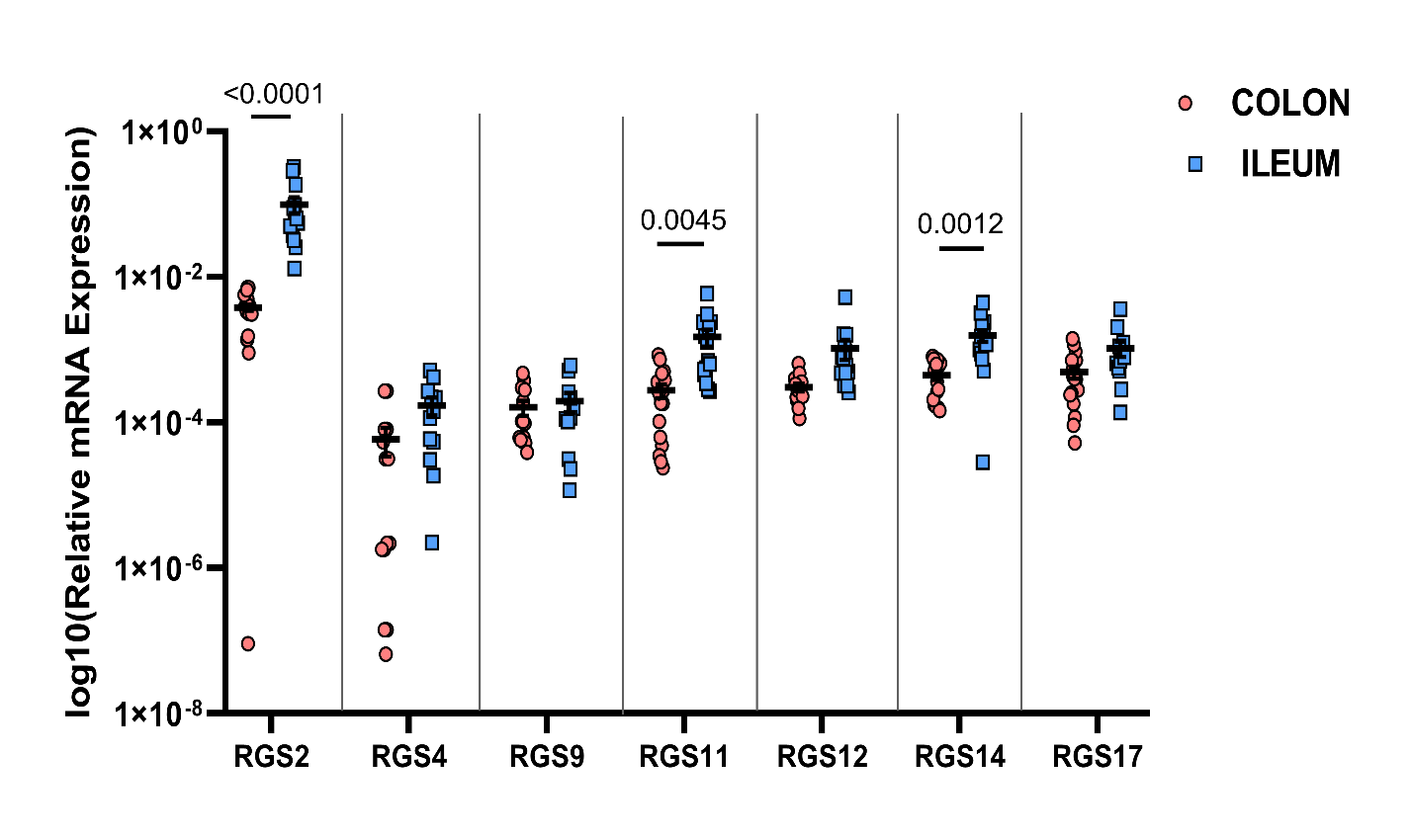
**

**Supplemental Figure 2.** Validation study completed in a separate cohort of *RGS* expression in the human colon (pink circle), and ileum (blue squares) in all participants (n=15)regardless of BMI status. Data showing individual values within a group include a vertical error bar line, denoting SD, and a horizontal line denoting Mean value.

**
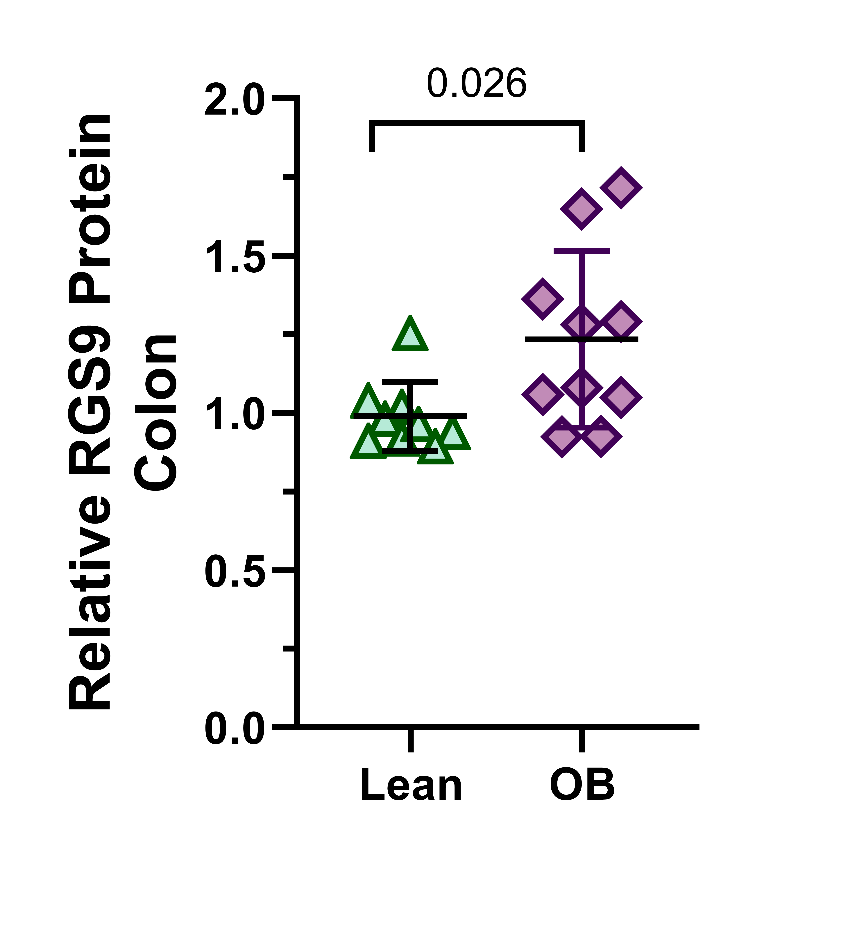
**

**Supplemental Figure 3.** Relative protein expression of colonic *RGS9* between obesity (n=10, purple diamonds) compared to lean (n=9, green triangles) using semiquantitative IHC/IF. Data showing individual values within a group include a vertical error bar line, denoting SD, and a horizontal line denoting Mean value.

| Supplemental Table 1. Human PCR primers used for RTqPCR gene expression studies | | | |
| --- | --- | --- | --- |
| Gene | NCBI Ref Sequence | Set | Sequence (5'-3') |
| *EEF2* | NM_001961.3 | Forward | TCCCCATCAAGAAATCTGACCC |
|  |  | Reverse | TTGTGCTTGTTGGGGGACTT |
| *GCG* | NM_002054.4 | Forward | GCCTTGGTGCAGAAGTACAGA |
|  |  | Reverse | TTCTGCTGTCTTCTGGTAGTGTG |
| *RGS2* | NM_002923.4 | Forward | TCCTCTACTCCTGGGAAGCC |
|  |  | Reverse | AGCAAGACCATATTTGCTGGCTA |
| *RGS4* | NM_001102445.2 | Forward | CGCAGGCATGTGAAGGAGAAA |
|  |  | Reverse | ATAAGCCCGGCAGCATACAGA |
| *RGS9* | NM_001081955.3 | Forward | ATGCCCTCCTGGAATGGACA |
|  |  | Reverse | ACAGCAACGACTGTTTGTTTCTTG |
| *RGS12* | NM_002926.3 | Forward | TGTCTGATGGCGAGTTGACG |
|  |  | Reverse | CAGGCGCTCAAAGGACACG |
| *PYY* | NM_004160.5 | Forward | GACGCCTACCCCATCAAACC |
|  |  | Reverse | TCTCTTTTCCCATACCGCTGC |

| Supplemental Table 2. Differential expression analysis of the EEC cluster or only *GCG+* L-type EECs for RGS family genes comparing obesity to lean from scRNA-Seq dataset. lean patients (n=4), and patients with obesity (n=5). mRNA expression reported as reported as Log_2_FC. | | | | |
| --- | --- | --- | --- | --- |
| GENE | All EECs  Log_2_FC  Ob vs. L | p-value | *GCG+* L-cells Log_2_FC  Ob vs. L | p-value |
| *RGS1* | 0.390 | 0.98 | -0.082 | 0.48 |
| *RGS2* | -0.750 | 0.04 | 0.412 | 0.32 |
| *RGS4* | -0.422 | 0.01 | 0.005 | 0.66 |
| *RGS5* | -0.013 | 0.65 | 0.040 | 0.24 |
| *RGS7* | 0.212 | 0.33 | 0.352 | 0.59 |
| *RGS9* | 0.287 | 0.19 | 0.609 | 0.10 |
| *RGS10* | 0.139 | 0.66 | 0.142 | 0.85 |
| *RGS11* | -0.109 | 0.91 | 0.218 | 0.73 |
| *RGS12* | 0.312 | 0.0019 | 0.320 | 0.20 |
| *RGS14* | 0.092 | 0.21 | 0.387 | 0.08 |
| *RGS19* | -0.013 | 0.48 | -0.062 | 0.48 |

**SUPPLEMENTARY REFERENCES**

1. McRae A, Ricardo-Silgado ML, Liu Y, Calderon G, Gonzalez-Izundegui D, Rohakhtar FR, et al. A Protocol for the Cryopreservation of Human Intestinal Mucosal Biopsies Compatible With Single-Cell Transcriptomics and Ex Vivo Studies. Frontiers in Physiology. 2022;13.

2. Roberts GP, Larraufie P, Richards P, Kay RG, Galvin SG, Miedzybrodzka EL, et al. Comparison of Human and Murine Enteroendocrine Cells by Transcriptomic and Peptidomic Profiling. Diabetes. 2019;68(5):1062-72.

3. Ersahin T, Carkacioglu L, Can T, Konu O, Atalay V, Cetin-Atalay R. Identification of novel reference genes based on MeSH categories. PLoS One. 2014;9(3):e93341.

4. Eissa N, Hussein H, Wang H, Rabbi MF, Bernstein CN, Ghia JE. Stability of Reference Genes for Messenger RNA Quantification by Real-Time PCR in Mouse Dextran Sodium Sulfate Experimental Colitis. PLoS One. 2016;11(5):e0156289.

5. Reimann F, Habib AM, Tolhurst G, Parker HE, Rogers GJ, Gribble FM. Glucose sensing in L cells: a primary cell study. Cell Metab. 2008;8(6):532-9.

6. Psichas A, Tolhurst G, Brighton CA, Gribble FM, Reimann F. Mixed Primary Cultures of Murine Small Intestine Intended for the Study of Gut Hormone Secretion and Live Cell Imaging of Enteroendocrine Cells. J Vis Exp. 2017(122).

7. Hayes MP, Bodle CR, Roman DL. Evaluation of the Selectivity and Cysteine Dependence of Inhibitors across the Regulator of G Protein-Signaling Family. Mol Pharmacol. 2018;93(1):25-35.
